# The EBV Immunoevasins vIL-10 and BNLF2a Protect Newly Infected B Cells from Immune Recognition and Elimination

**DOI:** 10.1371/journal.ppat.1002704

**Published:** 2012-05-17

**Authors:** Simon Jochum, Andreas Moosmann, Stephan Lang, Wolfgang Hammerschmidt, Reinhard Zeidler

**Affiliations:** 1 Research Unit Gene Vectors, Helmholtz Center, Munich, Germany; 2 Clinical Cooperation Group Immunooncology, Helmholtz Center, Munich, Germany; 3 Department of Otorhinolaryngology, Universitätsklinikum Essen, Essen, Germany; 4 Ludwig-Maximilians-Universität, Department of Otorhinolaryngology, Munich, Germany; Cambridge University, United Kingdom

## Abstract

Lifelong persistence of Epstein-Barr virus (EBV) in infected hosts is mainly owed to the virus' pronounced abilities to evade immune responses of its human host. Active immune evasion mechanisms reduce the immunogenicity of infected cells and are known to be of major importance during lytic infection. The EBV genes *BCRF1* and *BNLF2a* encode the viral homologue of IL-10 (vIL-10) and an inhibitor of the transporter associated with antigen processing (TAP), respectively. Both are known immunoevasins in EBV's lytic phase. Here we describe that *BCRF1* and *BNLF2a* are functionally expressed instantly upon infection of primary B cells. Using EBV mutants deficient in *BCRF1* and *BNLF2a*, we show that both factors contribute to evading EBV-specific immune responses during the earliest phase of infection. vIL-10 impairs NK cell mediated killing of infected B cells, interferes with CD4+ T-cell activity, and modulates cytokine responses, while BNLF2a reduces antigen presentation and recognition of newly infected cells by EBV-specific CD8+ T cells. Together, both factors significantly diminish the immunogenicity of EBV-infected cells during the initial, pre-latent phase of infection and may improve the establishment of a latent EBV infection *in vivo*.

## Author Summary

Despite strong cellular and humoral immune responses, herpesviruses persist in their hosts for a lifetime. Epstein-Barr virus (EBV) is a herpesvirus that infects human B cells. This results in a latent infection where only a minimal set of viral proteins is expressed and infected cells cannot be eradicated by immune cells. When the virus reactivates in order to produce progeny, many viral proteins are expressed that are potential targets of immunity, but the virus coexpresses viral “immunoevasins” that blunt immune responses. Similarly, in the very first phase of B cell infection by EBV, called the pre-latent phase, a rather wide spectrum of antigens is expressed. However, it has been unknown whether viral immunoevasion occurs in this phase. Here we show that two viral immunoevasins are active in the pre-latent phase and prevent immune recognition by a variety of mechanisms: they reduce the presentation of EBV antigens to CD8+ killer T cells, prevent an attack by natural killer cells, and reduce the function of CD4+ helper T cells. Thus, it seems to be important for the virus to shield itself from attack by immune cells during the pre-latent stage.

## Introduction

Epstein-Barr virus (EBV) is a ubiquitous human herpes virus with strong tropism for human B cells. EBV persists in an infected host for life by establishing a latent infection in B cells that represent an immunologically silent reservoir. Eventual reactivation of these cells into the lytic cycle leads to the production of progeny viruses that spread to other cells and hosts. The lytic phase goes along with the expression of high amounts of viral antigen, including the highly immunogenic immediate early proteins BZLF1, BRLF1 and BMRF1 [1]. To protect lytically activated cells from immune recognition, EBV takes several measures to perturb anti-viral immune responses of the host ([2] for review). EBV also expresses a set of immunogenic latent and lytic proteins immediately following the infection of target cells [3], [4], suggesting that also newly infected cells are prone to immune recognition. Hypothetically, the virus copes with this immunological challenge by active immune evasion in newly infected cells, which would be in close analogy to cells in the productive lytic phase.

EBV codes for a number of proteins that subvert the host's immune surveillance ([5] for review): a homologue of human IL-10 with anti-inflammatory properties (vIL10) [6]–[8], encoded by the EBV gene *BCRF1* [9], the DNAse/exonuclease BGLF5 that shuts off host protein synthesis [10] and contributes to Toll-like receptor 9 downregulation in productively infected cells [11], the G-protein-coupled receptor BILF1 that degrades MHC class I molecules [12], and BNLF2a, a protein unique to lymphocryptoviruses that inhibits the transporter associated with antigen processing (TAP) [13]. These proteins have so far been classified as lytic proteins and, correspondingly, were functionally investigated in lytic subsets of EBV-infected cell lines in vitro.

Several viruses including EBV, primate cytomegaloviruses (CMVs), Orf poxvirus, and equine herpes virus type 2 (EHV-2) encode homologues of human IL-10 [9], [14], [15], strongly suggesting that IL-10 is advantageous for these viruses. Accordingly, different immunomodulatory activities in infected and bystander cells have been described for viral IL-10 homologues including inhibition of DC maturation [16] and inhibition of Th1 cytokine expression [17]. Recently, it has been reported that vIL-10 of CMV has a profound impact on innate and adaptive immune responses in an in vivo model [18] EBV's IL-10 homologue has been described to be critical for B-cell growth transformation [6], to block gamma interferon release [19], to reduce MHC-I levels on B cells [8] and to functionally inhibit T cells [20] and monocytes [7]. vIL-10 encoded by *BCRF1* is expressed early upon infection [19], but its precise role and immunomodulatory capacities especially during the initial phase of EBV infection remain elusive.

BNLF2a, in contrast, is unique to the family of lymphocryptoviruses, but many other viruses pursue analogous strategies of TAP inhibition ([21] for review). BNLF2a prevents binding of both ATP and peptide to TAP and thereby prevents peptide loading to MHC class I molecules [22]. Ectopic expression of BNLF2a leads to reduced surface levels of MHC class I molecules [13] that are unstable without properly loaded peptides [23]. BNLF2a is expressed early in the productive lytic phase and reduces the recognition of B cells by T lymphocytes specific for viral immediate early and early lytic proteins [24].

In this study, we extend our knowledge about BNLF2a and vIL-10/BCRF1. We show that both proteins contribute to the immune evasion of EBV in newly infected primary B cells. Both proteins are expressed immediately following infection. With EBV mutants deficient in *BCRF1* and *BNLF2a*, we demonstrate that BNLF2a impairs the recognition of virally infected B cells by EBV-specific CD8+ T lymphocytes during the very first days of infection. Additionally, we identified vIL-10 to protect EBV-infected B cells from NK cell-mediated elimination. Furthermore, vIL10 released from newly infected B cells prevents the secretion of anti-viral cytokines, thereby abrogating anti-viral CD4+ effector T cell functions. In summary, BNLF2a and vIL-10/BCRF1 act in a complementary manner to prevent immune recognition and elimination of newly EBV-infected B cells.

## Results

### Construction of *BCRF1*- and *BNLF2a*-deficient maxi-EBV genomes

Maxi-EBV genomes deficient in *BCRF1* and/or *BNLF2a* were constructed by targeted mutation of the maxi-EBV plasmid p2089 [25]. Maxi-EBV mutagenesis was performed by homologous recombination in accordance to previous work [26]. *BCRF1* deletion mutants were generated by replacing the entire gene by a prokaryotic kanamycin resistance expression cassette. The *BNLF2a* locus of EBV is complex (Figure S1A). *BNLF2a* shares its transcript with *BNLF2b* and is situated in the first intron of the TP gene encoding the latent membrane protein (LMP) 2A. Moreover, this genomic locus is part of the 3′ untranslated region of BNLF1 encoding LMP1. To abrogate BNLF2a expression, the first translational start codon of *BNLF2a* was mutated to a stop codon preventing BNLF2a translation. The exchange of only four nucleotides reduced the risk of interfering with the expression or regulation of adjacent genes. In total, we constructed three EBV mutants: two single mutants that were null for BCRF1 (ΔBCRF1) or BNLF2a (ΔBNLF2a) and one double mutant that combined both functional deletions (double k.o.). Technical details, cloning strategies, and restriction enzyme digests confirming BAC integrity are provided in Material & Methods and Figure S1.

**Figure 1:**
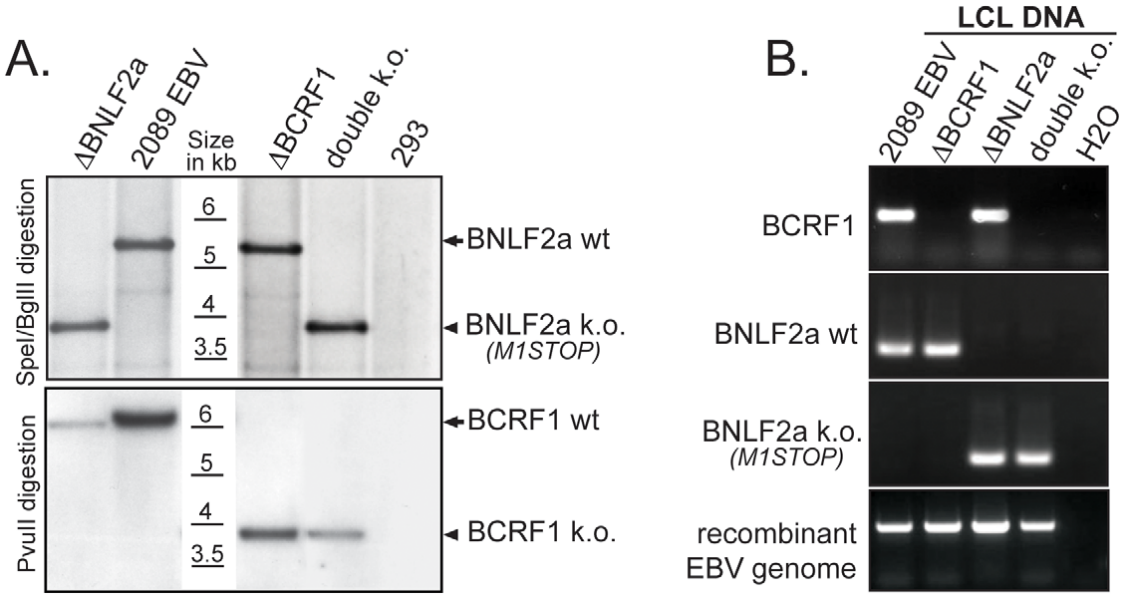
Generation of mutant viruses. (A) Southern blot hybridizations were performed with digested genomic DNA of 293HEK-derived virus producer cells using radioactive probes complementary to genomic regions adjacent to the modified loci. The different band intensities are due to slightly different amounts of DNA loaded onto the gels. (B) Lymphoblastoid cell lines (LCLs) were generated by infecting primary B cells with recombinant viruses. The viral genotype in these LCLs was assessed by PCR. *BCRF1* deletion was confirmed by absence of signal, the *BNLF2a* genotype was determined with primer pairs that specifically detect wild-type *BNLF2a* sequences or the mutated 4 nucleotides of *ΔBNLF2a*.

We established single cell clones from HEK293 cells stably transfected with the mutant viruses described above by selecting for hygromycin resistance. Clonal cells lines were tested for virus production upon transfection of an expression plasmid encoding the lytic transactivator BZLF1 [27]. The titers of infectious virus in the supernatants of these clones were calculated as described in Material & Methods. The genotypes of selected clones were confirmed by Southern blot hybridization (Figure 1A) and infected B cells were routinely tested by PCR to confirm infection with the respective virus mutant (Figure 1B).

### BCRF1 and BNLF2a are expressed by day one of infection

**Figure 2:**
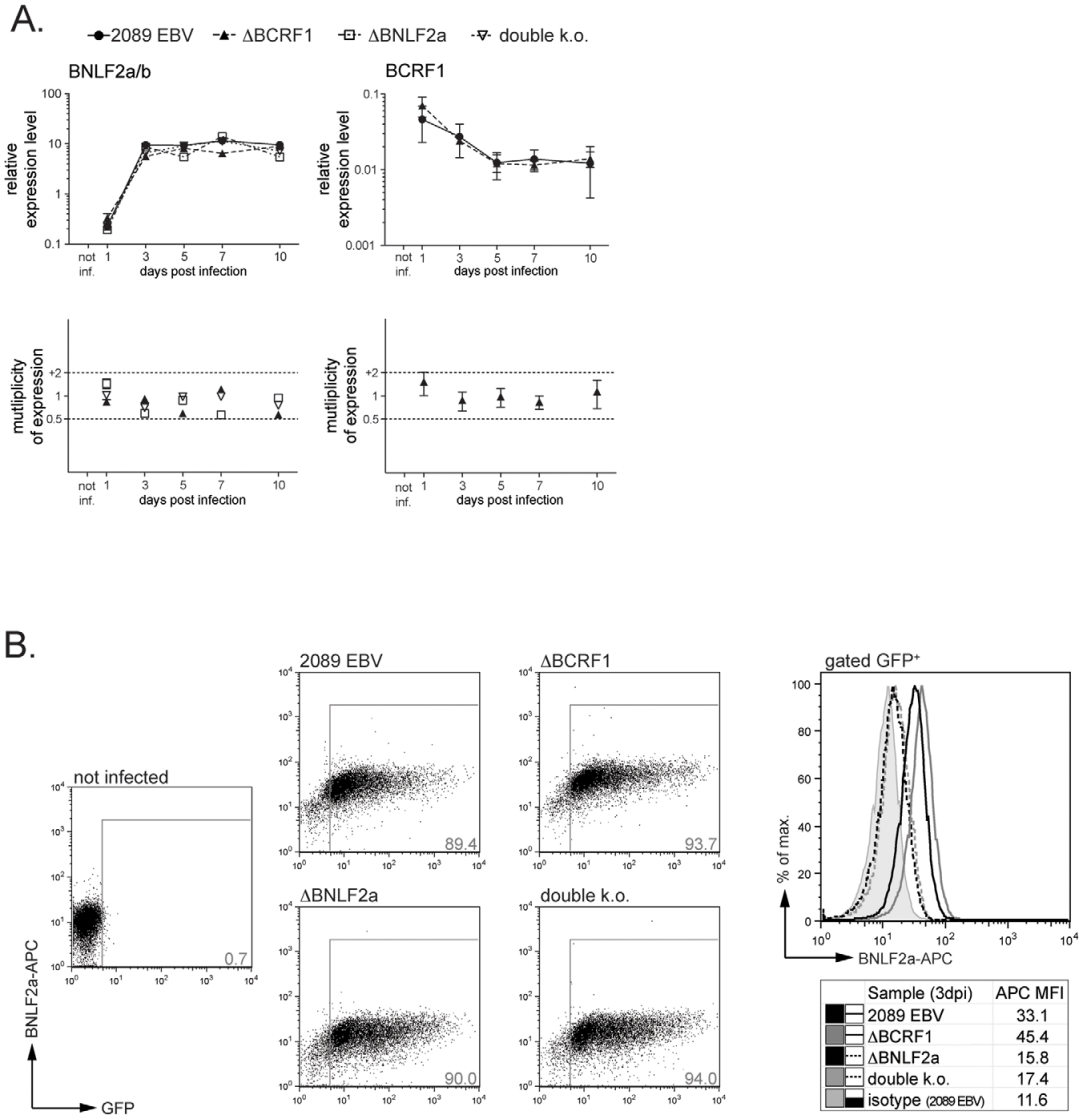
The immunoevasins vIL-10 and BCRF1 are expressed during the initial phase of infection. (A) Peripheral B cells were infected with 2089 wild-type EBV or mutant viruses and total RNA was isolated at the indicated time points. The transcript levels of BCRF1 and BNLF2a/b were assessed by quantitative RT-PCR and are shown in relation to transcripts levels of the housekeeping gene *glucuronidase beta* (*gusb*). Note that BNLF2a and BNLF2b are encoded on the same bicistronic transcript and cannot be distinguished by PCR. Transcripts of BNLF2a/b are present in ΔBNLF2a- and double k.o.-infected B cells, but BNLF2a translation is abrogated (see also Figure 2B and Figure S1). Relative transcript levels in mutant virus-infected cells were normalized to those of 2089 EBV-infected B cells and values are shown as multiplicity of expression in the lower panel. (B) B cells infected with 2089 EBV or ΔBCRF1 mutant virus express BNLF2a protein, whereas no BNLF2a protein could be detected in cells infected with ΔBNLF2a- and double k.o mutant viruses. B cells were infected with the indicated viruses and analyzed for presence of BNLF2a protein expression by intracellular flow cytometry at day 3 p.i. Histograms show gated GFP+, i.e. infected cells. MFI = mean fluorescence intensity.

EBV expresses a set of lytic genes during the initial, pre-latent phase of B-cell infection [3], [4] and EBV virions contain a variety of viral RNAs [28], which prompted us to address the expression kinetics of the immunomodulatory proteins vIL-10/*BCRF1* and BNLF2a during pre-latent infection. For this, we infected primary peripheral B cells with 2089 wild-type EBV or with the ΔBNLF2a, ΔBCRF1 or double k.o. mutant viruses. We then prepared cDNA from infected cells at different time points post infection (p.i.) and assessed the expression of the *BCRF1* gene as well as levels of the bicistronic transcript encoding BNLF2a and BNLF2b by quantitative RT-PCR (qPCR). Figure 2A shows that both transcripts were detectably present as early as one day p.i. The comparison to glucuronidase beta (GUSB) transcripts, a validated housekeeping gene in LCLs [29], revealed that BNLF2a/b expression levels increased initially, followed by a plateau, whereas BCRF1 transcript levels declined during the first days p.i. before reaching a stable level. Performing flow cytometry, we could demonstrate the rapid expression of BNLF2a protein in cells infected with 2089 wild-type EBV and ΔBCRF1 mutant EBV, but not in cells infected with ΔBNLF2a or double k.o. mutant viruses (Figure 2B) confirming the genetic ablation of *BNLF2a*. No specific vIL-10-antibody was available to confirm *BCRF1* deficiency.

### BNLF2a reduces the recognition of infected cells by EBV-specific CD8+ T cell clones

BNLF2a interferes with antigen presentation on MHC class I molecules by inhibiting the transporter associated with antigen processing (TAP) [13], [22]. EBV-specific CD8+ T-cell clones constitute sensitive tools to measure antigen presentation of EBV-infected B cells in vitro. In order to analyze BNLF2a effects during the earliest phase of infection, we infected primary peripheral B cells with 2089 wild-type EBV or with the mutant viruses ΔBCRF1, ΔBNLF2a, or double k.o. and used them as targets for clonal CD8+ T cells. One of these T-cell clones detects the HLA-B8-restricted epitope RAKFKQLL (RAK) derived from BZLF1 protein [30], the master regulator of the lytic cycle [31]. Co-cultures at defined effector/target ratios were prepared at different days after B-cell infection and incubated overnight. ELISA assays on gamma interferon (IFN-γ) levels in the supernatant were indicative of T-cell activation.

**Figure 3:**
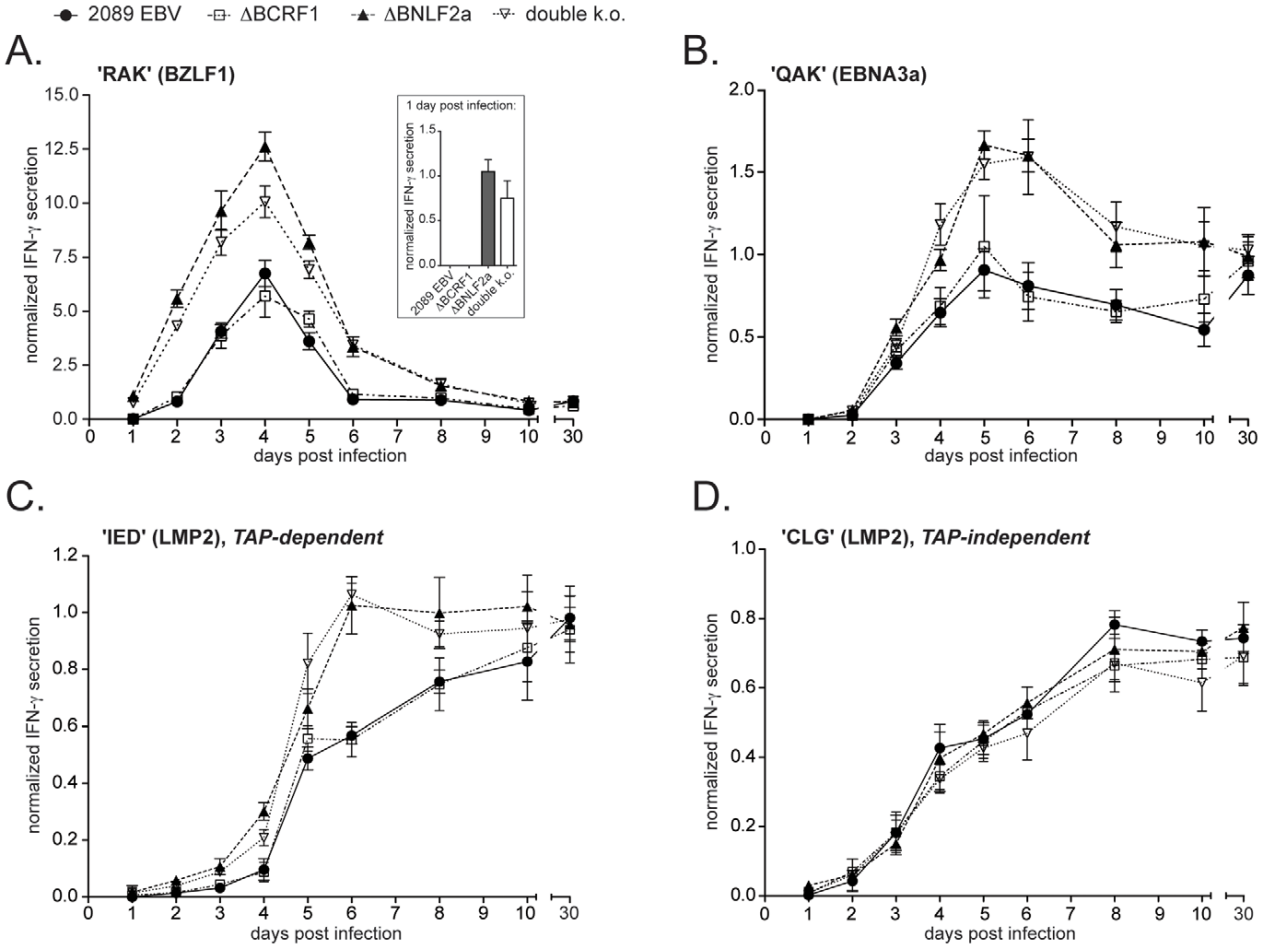
vIL-10 impairs recognition of newly infected B cells by clonal EBV-specific CD8+ T cells in a TAP dependent manner. Co-cultures with constant ratios of clonal EBV-specific CD8+ T cells and HLA-matched B cells infected with 2089 EBV, ΔBNLF2a, ΔBCRF1, or double k.o. virus were prepared at the indicated day post infection and incubated for 18 hours. IFNγ concentrations in the culture supernatant were assessed by ELISA. Values were normalized to the recognition of a 2089 EBV infected, established LCL to correct for variable activities of the T cell clone on the respective day. These reference samples contained IFN-γ levels of 227–343 pg/ml (RAK), 1261–1577 pg/ml (QAK), 684–891 pg/ml (IED) and 1034–1157 pg/ml (CLG). Error bars indicate the standard deviation of three replicates, the significance of difference was calculated by two way ANOVA analysis, ** p<0.01 *** p<0.001. Recognition assays were performed with clonal EBV-specific CD8+ T cells specific for (A) the RAK epitope of BZLF1 protein, (B) the QAK epitope of EBNA3a protein, (C) the TAP-dependent IED epitope of LMP2 and (D) the TAP-independent CLG epitope of LMP2.

The experiments revealed that RAK-specific T cells recognized B cells infected with either ΔBNLF2a or the double k.o. mutant virus significantly better than B cells infected with either 2089 wild-type or ΔBCRF1 mutant EBVs (Figure 3A). Of note, the difference in recognition became evident already on day 1 p.i. At this time point, only B cells infected with ΔBNLF2a or double k.o. mutant viruses detectably activated the RAK-specific T cell clone, whereas cells infected with 2089 EBV or the ΔBCRF1 mutant virus did not. The level of recognition of infected B cells peaked on day 4 p.i. and then declined until it reached similar levels as in control LCLs, indicating the establishment of latency. Similar results were obtained with CD8+ T-cell clones specific for the epitopes QAKWRLQTL (QAK) (Figure 3B) and IEDPPFNSL (IED) (Figure 3C) derived from the latent proteins EBNA3a and LMP2a, respectively, further emphasizing the immunoevasive function of BNLF2a in freshly infected cells. In contrast, the response of clonal CD8+ T cells specific for the CLG epitope of LMP2 protein was independent of BNLF2a (Figure 3D). This epitope is known to be loaded TAP-independently onto MHC I molecules because its high hydrophobicity presumably allows for passive diffusion through the ER membrane [32]. The delay in T-cell recognition as compared to gene expression is presumably attributable to the fact that EBV-infected B cells reach their full antigen presenting potential not until a few days p.i. [33] or to antigen immunodominance [34]. To ensure that our genetic manipulation did not alter expression levels of the investigated antigens, we performed quantitative PCR that revealed similar expression levels in B cells infected with the different viruses (Figure S2).

### vIL-10 thwarts the release of antiviral cytokines and NK/NKT cell mediated lysis

**Figure 4:**
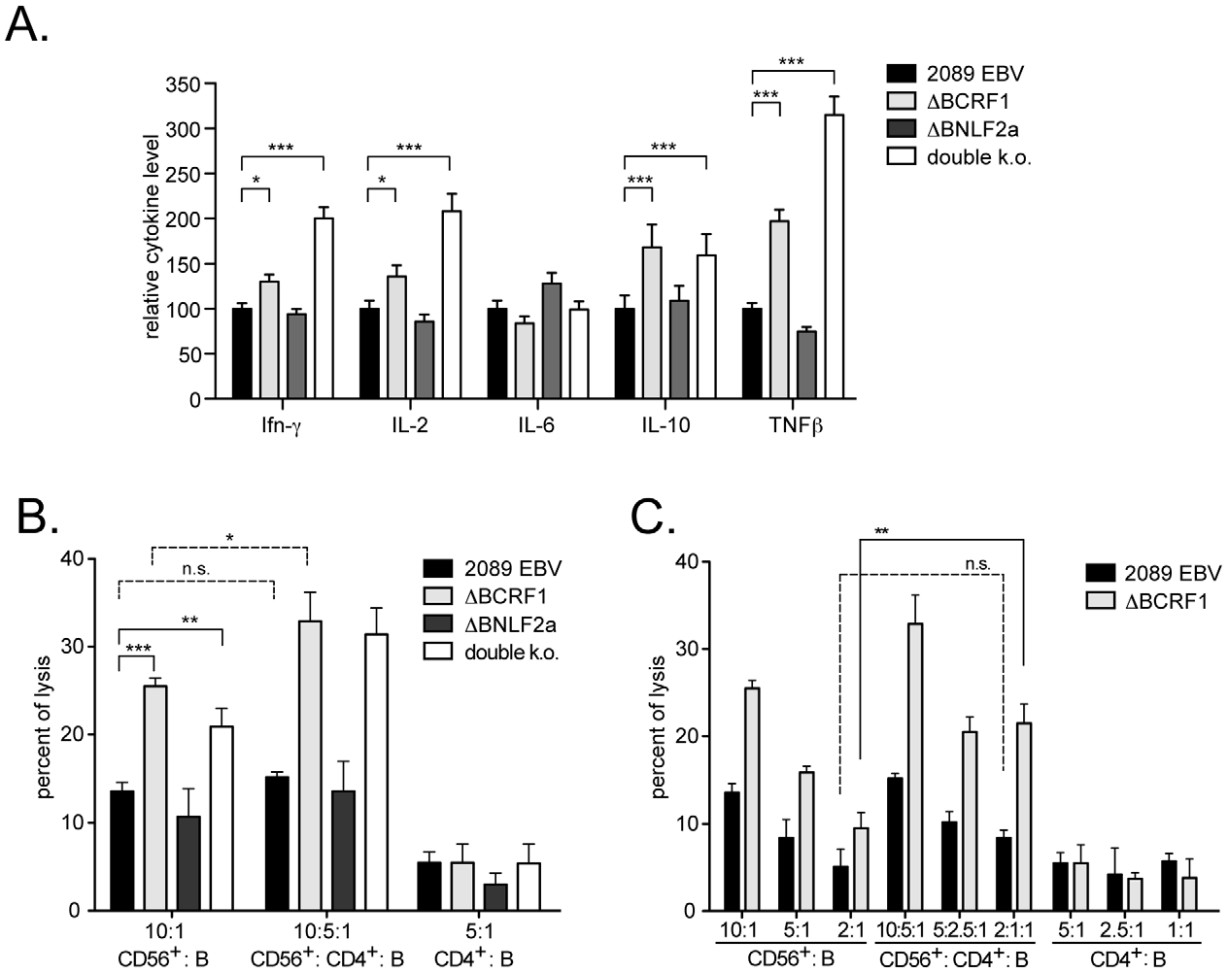
vIL-10 skews the inflammatory cytokine response of PBMCs to an EBV infection, protects EBV infected cells from NK/NKT-mediated lysis and subverts CD4+ T cell support. (A) PBMCs of an EBV-seropositive donor were infected with 2089 EBV, ΔBCRF1, ΔBNLF2a, or double k.o. virus and cultured for 9 days. The Th1/Th2 cytokine response of these cultures was assessed by multiplex-ELISA and values were normalized to levels of 2089 EBV infected samples. Error bars indicate the standard deviation of three replicates. (B) Peripheral B cells were infected with 2089 EBV, ΔBCRF1, ΔBNLF2a, or double k.o. virus and labeled with calcein 3 days p.i. Autologous CD56+ cells and CD4+ cells were isolated from PBMCs, added to the labeled B cells at the indicated ratios and incubated for three hours. (C) Same experiment as shown in B with modified effector/helper/target ratios. Calcein fluorescence intensity of the supernatant was indicative of B cell lysis. Treatment of B cells with 1% Triton-X-100 led to complete lysis with maximum calcein release and samples were related to this value. Error bars indicate standard deviations of three replicates. The significance of difference was calculated by a two-way ANOVA, *: p<0.05, **: p<0.01, ***: p<0.001. The shown data are representative for three independent experiments.

The previous experiments revealed that vIL-10 did not influence the recognition of freshly infected B cells by EBV-specific CD8+ T cell clones in vitro (Figure 3). Nevertheless, its conservation in different EBV isolates [35] and the strong immunomodulatory capacity of its cellular homologue [36] both suggest a prominent role for *BCRF1*/vIL-10 in EBV infection. IL-10 is known to sustainably promote Th2 cytokine responses [36], which prompted us to assess the influence of vIL-10 on the secretion of various Th1/Th2 cytokines by PBMCs in response to an EBV infection. We prepared PBMCs from a donor with EBV immunity, determined the B cell content and infected them with 2089 EBV, ΔBCRF1, ΔBNLF2a, or double k.o. mutant viruses with 0.1 GRU/B cell. We cultured these EBV-infected PBMCs for twelve days and evaluated the levels of Th1 and Th2 cytokines in the supernatants by multiplex ELISAs. The cytokine composition in the supernatants differed between PBMCs infected with the *BCRF1*-positive viruses (2089 EBV, ΔBNLF2a) and mutant viruses lacking *BCRF1* (ΔBCRF1, double k.o.). In detail, PBMCs infected with the ΔBCRF1 or double k.o. mutant viruses produced significantly higher levels of the pro-inflammatory cytokines IFN-γ, IL-2, IL-6, and TNF-β and of anti-inflammatory IL-10 (Figure 4A), whereas levels of IL-1, IL-5, IL-8, and TNF-α were not affected (not shown). Interestingly, we observed the highest levels of the hIL-10 in the supernatants of PBMCs that were infected with either of the BCRF1-lacking mutant viruses ΔBCRF1 or double k.o. This observation points to the regulation of hIL-10 by vIL-10 or to increased IL-10 release in the course of a secondary cytokine response evoked by the high levels of pro-inflammatory cytokines.

Besides activated T cells, NK cells can specifically lyse virus-infected cells. Regarding EBV, NK cells preferentially eliminate infected cells in lytic phase [37]. To address the question whether NK/NKT cell-mediated lysis differed between B cells infected with 2089 wild-type or mutant EBVs in the pre-latent phase, we infected purified peripheral B cells and added autologous purified CD56+ NK/NKT cells on day 3 p.i. We then assessed specific B-cell lysis after 3 hours of co-incubation and observed a significantly stronger lysis of B cells infected with the ΔBCRF1 or double k.o. mutant viruses as compared to B cells infected with 2089 EBV or the ΔBNLF2a mutant virus (Figure 4B left panel).

In a parallel experiment, we included CD4+ T cells that represent an important source for many cytokines. We hypothesized that CD4+ T cells could provide a supporting microenvironment for NK/NKT cells. Indeed, we found that the presence of CD4+ T increased NK/NKT-mediated lysis of infected B cells. This adjuvant effect was most pronounced when B cells were infected with either ΔBCRF1 or double k.o. mutant viruses (Figure 4B mid panel). The difference became most evident at lower effector/helper/target ratios (Figure 4C). In contrast, CD4+ T cells did not significantly increase NK/NKT-mediated lysis of B cells with 2089 wild-type or ΔBNLF2a mutant EBV (Figure 4B, middle panel). CD4+ T cells alone did not reveal any cytolytic activity above background (Figure 4B, right panel).

The cytolytic activity of NK cells is regulated by MHC class I levels and by accessory molecules such as ligands of the activating natural killer group 2 member D (NKG2DL), comprising MICA, MICB, and UL16-binding proteins (ULBP) 1–6. Since IL-10 was described to modulate MHC surface levels [8] and expression of NKG2D ligands [38], we investigated the expression of these molecules in more detail. Flow cytometry revealed that newly infected B cells displayed different levels of MHC class I at their surfaces varying in a temporal fashion throughout the first days of infection (Figure S3A). However, this pattern of MHC I surface levels was not affected by the use of 2089 EBV or mutant virus. MHC I/BNLF2a double staining of GFP-positive, i.e. infected B cells 3 days p.i. particularly correlated that endogenous expression levels of BNLF2a did not alter the surface levels of MHC class I molecules of cells infected with different viruses (Figure S3B and C). Hence, improved NK killing of B cells infected with either ΔBCRF1 or double k.o. mutant EBVs was not attributable to different MHC class I levels. Next, we analyzed the expression levels of members of the family of NKG2D ligands by qPCR. With the exception of ULBP4 and 6, all ligands were clearly expressed. Importantly, expression kinetics were independent of the virus mutant used for infection (Figure S4). As NK and CD4+ T cells express the IL-10R ([39] and Figure S5) our data suggested a direct effect of vIL-10 on these effector cells. This hypothesis was further substantiated by rescue experiments: the addition of exogenous viral and human IL-10 at physiological concentrations (1 ng/ml) partially reverted NK-mediated killing of infected B cells, and exogenous vIL-10 completely reverted CD4+ T cell assistance (Figure S6). K562 cells do not express MHC I molecules and are therefore efficiently killed by NK/NKT cells. Intriguingly, the same concentration of IL-10 that inhibited killing of newly EBV-infected B cells did not affect NK/NKT-mediated killing of K562 cells (Figure S7). Taken together, our results indicate that vIL-10 directly impairs NK/NKT-mediated lysis of newly EBV-infected B cells and inhibits CD4+ T cell support of NK-mediated killing.

### Deletion of both *BNLF2a* and *BCRF1* improves long-term immune control of infected B cells *in vitro*

**Figure 5:**
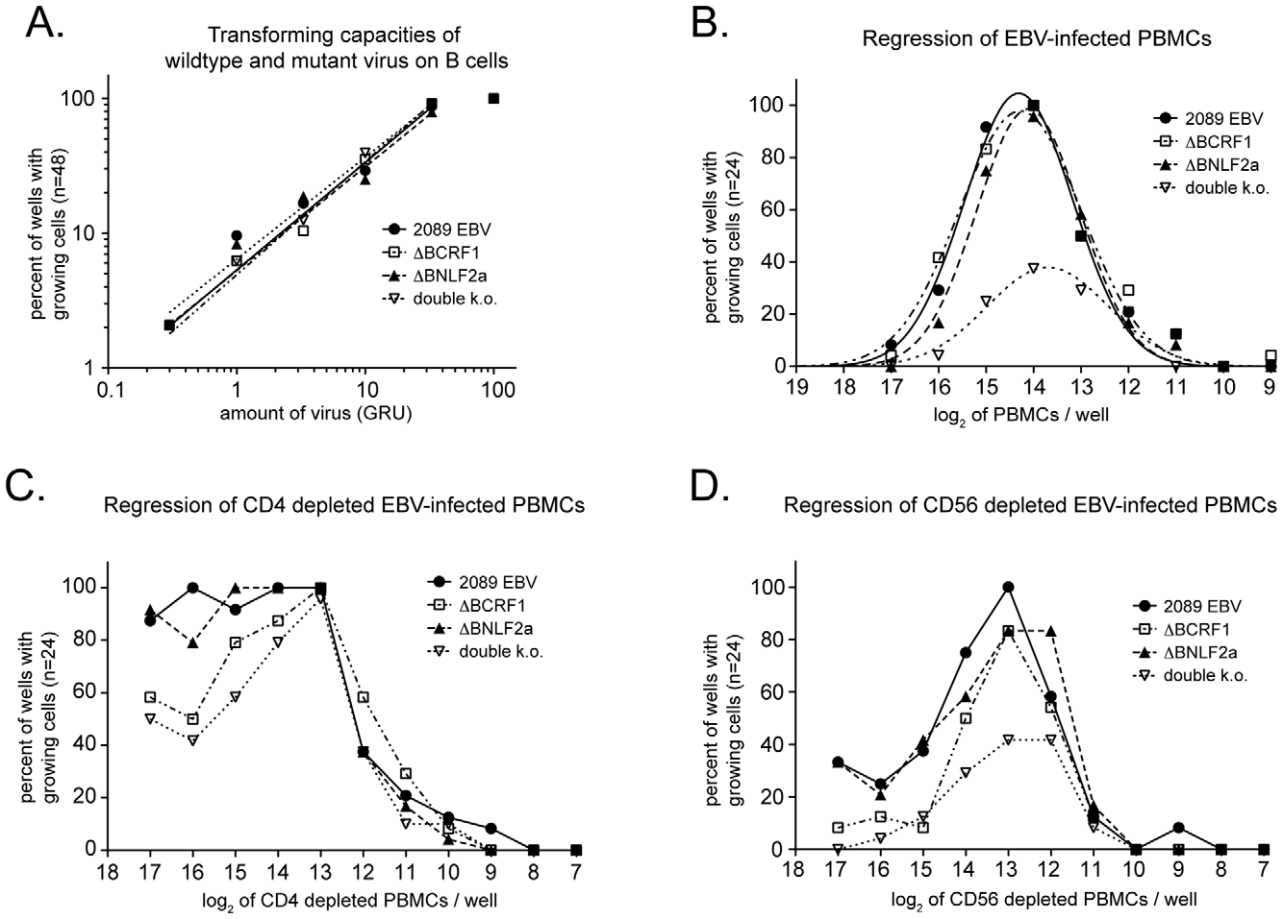
Deletion of *BNLF2a* and *BCRF1* leads to improved long-term immune control of infected B cells. (A) 2089 EBV, ΔBCRF1, ΔBNLF2a, and double k.o. viruses have identical transformation capacities. B cells were isolated from PBMCs and infected in 96-well plates with serial dilutions of the indicated virus stocks. 48 replicates of each dilution were analyzed six weeks later and the number of wells with proliferating cells was determined in an MTT assay. (B) The regression of double k.o.-infected B cells is more efficient as compared to regression of cells infected with either 2089 EBV or the ΔBCRF1 and ΔBNLF2a single mutant viruses. PBMCs of an EBV-seropositive donor were infected with the indicated viruses and cultured for six weeks. The proportion of wells with proliferating cells is plotted against the log_2_ of the initial amount of plated cells/well. Logarithmic Gaussian distributions are fitted to the data sets (R^2^>0.95). (C) The incidence of regression of EBV-infected B cells was much weaker in CD4-depleted PBMCs. However, proliferation of B cells infected with either ΔBCRF1 or double k.o. mutant viruses was inhibited more than cells infected with either 2089 EBV or ΔBNLF2a mutant virus. (D) CD56+ NK/NKT cells contribute little to the regression of EBV-infected B cells, but B cells infected with ΔBCRF1 or double k.o. mutant EBVs were eliminated more efficiently.

Regression assays are a means to quantify EBV-specific memory T-cell responses in vitro. In such assays, experimentally infected PBMCs from EBV-positive donors show regression of B-cell outgrowth *in vitro*, reflecting the reactivation of an EBV-specific memory T-cell response [40]. The strength of regression depends on the number of EBV-specific immune effectors, which mirror the extent of the donor's EBV immunity as well as the ability of infected cells to escape immune elimination. Identical EBV-mediated transformation of primary B cells is a prerequisite when comparing different viruses in regression assays. Therefore, we initially determined the dose-dependent transformation of B cells by wild-type and mutant EBVs in limiting dilution assays. Purified peripheral B cells were infected with serial dilutions of the different virus stocks and the number of outgrowing lymphoblastoid cells was scored six weeks p.i. As shown in Figure 5A, we did not observe any differences in the rates of B-cell transformation. In a next series of experiments, we infected serial dilutions of PBMC preparations from EBV-seropositive donors with 2089 wild-type EBV or the ΔBCRF1, ΔBNLF2a, or double k.o. mutant viruses and seeded the cells in 96-well cluster plates. Six weeks after infection we analyzed cell viability in an MTT assay. B cells infected with ΔBCRF1, ΔBNLF2a, or wild-type EBV were killed equally but B cells infected with the double k.o. mutant EBV were eradicated much more efficiently (Figure 5B). Thus, deletion of vIL-10 and BNLF2a synergistically affected the outgrowth of infected B cells in the presence of EBV-specific immune effectors in vitro. This finding strongly suggests that pre-latent vIL-10- and BNLF2a-mediated immune evasion contributes to the success of EBV infection also in its native host.

In line with results published by others [41]–[43], subsequent analyses with CD4-depleted PBMCs indicated that CD4+ T cells are essential for regression of EBV-infected B cells in vitro (Figure 5C). In this setting, immune control of B cells infected with 2089 wild-type or ΔBNLF2a mutant EBVs was completely abrogated, while regression of B cells infected with either ΔBCRF1 or double k.o. mutant virus was partially maintained. These results suggested that helper CD4+ T cells contribute to regression, probably by providing stimulatory cytokines such as IL-2 to CD8+ effector T cells [41], and vIL-10 directly interferes with reactivation of EBV-specific memory CD8+ T-cells. Consistent with published data [41], the contribution of CD56+ NK/NKT cells to the regression of EBV-infected PBMCs was less pronounced, but immune control of cells infected with 2089 wild-type or ΔBNLF2a mutant EBVs was slightly reduced in comparison to non-depleted PBMCs, which is again in line with a potential inhibitory effect of vIL-10 on CD4+ or CD8+ T cells (Figure 5D).

## Discussion

EBV persists for life in its host despite the presence of strong anti-viral immune responses. The asymptomatic co-existence depends on an immunological equilibrium of anti-viral activities of the host and viral counter-mechanisms. Thus, reduced viral protein expression during latency as well as active immune evasion is essential for EBV. Recently, a number of viral strategies of active immune evasion during the lytic phase have been identified for EBV ([5] for review). Especially immediate-early and early lytic proteins are among the most immunodominant EBV antigens [44] rendering their expression a particular immunological challenge for the virus. Recent reports described that immediate-early and early lytic genes are also expressed in newly infected cells following infection [3], [4] putting these cells at risk for rapid elimination by immune effectors. Current data from our lab revealed that EBV virions deliver viral mRNAs, including those encoding the immunoevasins vIL-10 and BNLF2a, into target cells where they are instantly translated [28]. Along this line, our findings demonstrate that specific CD8+ T cells can recognize EBV infection of B cells as soon as one day p.i. Our observation that the RAK epitope of BZLF1 is presented instantaneously after infection fits the data of others on early BZLF1 expression in newly infected cells [3], [4]. The immediacy of its expression may contribute to the observed immunodominance of BZLF1 among CD8+ target antigens of EBV [34]. This immunodominance may be shaped by cross-competition between CD8+ T cells for antigen-presenting cells, a process that favors T-cell responses against the earliest antigens presented during the process of infection [45].

EBV expresses the immune modulators vIL-10 and BNLF2a as early as six hours p.i. and actively perturbs the host's immune response to newly infected cells. These findings further indicate that the pre-latent phase of EBV is critical for the outcome and success of viral infection. Of note, our T-cell experiments confirm that BNLF2a blunts activation of EBV-specific CD8+ T cells as early as one day p.i. As BNLF2a was reported to interfere with MHC peptide loading, destabilized MHC results in reduced surface levels that might render the infected cells vulnerable towards NK cells. However, B cells infected with 2089 wild-type EBV or ΔBNLF2a mutant virus were equally lysed by NK/NKT cells (Figure 3B) and displayed similar MHC class I surface levels (Figure S3). Hence, expression of BNLF2a seems to be tightly balanced in that it impairs loading of new antigenic peptides without reducing MHC-I surface levels during the pre-latent phase of infection.

Expression of vIL-10 did not impair recognition of EBV-infected cells by clonal, EBV-specific CD8+ T cells (Figure 2). Cultured T-cell clones are pre-activated and the endogenous expression level of vIL-10 might be insufficient to repress these effectors. However, we detected direct effects of vIL-10 on ex vivo-isolated NK/NKT cells as well as CD4+ T cells. NK cells lyse EBV-infected B cells preferentially when they enter the productive lytic cycle [34]. We demonstrate here that NK cells also lyse newly infected B cells but vIL-10 interferes with this effector function (Figure 4). The presence of CD4+ T cells further supported NK-mediated target lysis, especially when vIL-10 was not expressed. This phenomenon is probably attributable to two different observations: (i) infections with *BCRF1*-deficient viruses led to higher levels of Th1 cytokines (Figure 4A) suggesting that increased Th1 cytokine secretion by CD4+ T cells boosted NK cell activity, and (ii) rescue experiments indicated a direct inhibitory effect of vIL-10, as well as hIL-10, on NK and CD4+ T cell activity (Figure 4B).

Our observation that BNLF2a and vIL-10 reduce the recognition of pre-latently infected B cells in the absence of MHC class I downregulation is in apparent contrast to earlier results pointing to downregulation of MHC class I by BNLF2a [13] or by vIL-10 [8]. It appears that BNLF2a can strongly decrease total MHC class I levels when ectopically expressed at high levels [13], weakly so when endogenously expressed in lytically EBV-infected cells [24], and not to a detectable extent when transiently expressed in the pre-latent phase (our present data). In lytically EBV-infected lymphoblastoid cells, the presentation of relevant EBV epitopes, including the BZLF1 RAK epitope, was reduced by BNLF2a much more strongly than were total MHC class I levels [24]. Thus, our observations that overall MHC class I levels are maintained in pre-latently infected cells, while T cell recognition is reduced, can be well reconciled.

Different considerations apply to the effects of vIL-10 on MHC class I levels. In our earlier study [8], we showed that exogenous soluble vIL-10 or human IL-10, as well as supernatants from the EBV-producing simian B cell line B95-8, reduced total MHC class I levels on primary human B cells after 2 days' incubation. EBV-containing B95-8 supernatant contains significant amounts of IL-10 (own observations). The conditions carried out with B95-8 supernatants might therefore mirror biological situations in secondary lymphoid organs in the amplification phase of infectious mononucleosis [46], [47]. Whether such high IL-10 concentrations are still beneficial for the onset of infection or rather trigger NK cell activity is however questionable. In contrast, the conditions in our present study (12 h incubation of primary B cells in infectious supernatant and subsequent exchange of media) potentially correspond to the conditions during acquisition of EBV from another virus carrier or spread of the virus for infection maintenance in the context of functional immune control. Our data demonstrate that pre-latent expression levels of endogenous viral IL-10 and BNLF2a together are balanced to avoid the reduction of MHC I surface levels in the pre-latent phase. Non-maximal expression necessarily results in more subtle effects, but complementarity of vIL-10 and BNLF2a might compensate for that. Hence, together both factors succeed to blunt cellular immune responses: while pre-latently expressed vIL-10 appears to act mainly on NK and T helper cells in a paracrine fashion, BNLF2a specifically blocks antigen presentation on MHC class I in infected cells.

The overall effects of the two immunoevasins BNLF2a and vIL-10 on establishment of EBV infection in a complex immunological environment were studied in regression assays. In this experimental setting, we observed a phenotype with the double k.o. mutant virus, only, revealing a synergistic effect between the two immunoevasins (Figure 5A). As viral functions within the first days of infection are decisive for the outcome of regression assays [48], our result further emphasizes the importance of BNLF2a and vIL-10. The experiments shown in Figure 5 also stress the role of CD4+ T cells in anti-viral immune responses, presumably by providing help to CD8+ cells [41]. Depletion of CD56+ NK cells had a minor effect, only, in accordance with previous observations [41]. This result and the observation that single ΔBNLF2a and ΔBCRF1 mutant viruses were as well controlled as wild-type EBV points at a degree of redundancy in immunological mechanisms of EBV control that can only be overcome by the combined action of viral immunoevasins with different mechanisms of action.

Collectively, we demonstrate in this study that the immunoevasins vIL-10 and BNLF2a of EBV have important functions in B cells in the pre-latent phase immediately following infection. Together, these proteins interfere both with innate and adaptive immune responses and thus contribute to efficient immune evasion of newly infected B cells. These findings highlight that the pre-latent phase of EBV infection is decisive for successful establishment and persistence of the virus in its host.

## Materials and Methods

### Ethics statement

The study was approved by the Ethics Committee of the Ludwig-Maximilians-Universität. Study participants or their legal guardians provided written informed consent.

### Construction of *ΔBCRF1* and *ΔBNLF2a* mutant viruses

The mutant viruses generated for this work are based on the previously described 2089 EBV [25]. The strategies to replace *BCRF1* and to block *BNLF2a* translation are depicted in Figure S1. The maxi-EBV genomes were modified following an improved protocol for BAC recombineering in the E.coli strain SW105 [26]. The *BCRF1* gene was replaced by a prokaryotic expression cassette for neomycin phosphotransferase II, conferring kanamycin resistance, flanked by homologous sequences (200 bp) to the neighboring region of the gene. The DNA fragment was cloned, linearized and electroporated into recombination competent SW105 bacteria carrying the 2089 EBV genome. Transformants were selected for kanamycin resistance (50 µg/ml). The first codon (Met1) of *BNLF2a* was replaced by a stop codon and concomitant insertion of an analytic SpeI site. First, a prokaryotic expression cassette for the galK gene flanked by 50 bp of homologous sequence to the nucleotides up- and downstream of the BNLF2a Met-1 codon was generated by PCR. The product was inserted into the 2089 EBV BAC by homologous recombination and SW105 clones were selected for competence in galactose metabolism. Then, a DNA fragment of 132 bp was synthesized comprising the four mutated nucleotides flanked by 64 bp of sequences homologous to the neighboring regions of the previously inserted galK cassette. Successfully modified clones were counter-selected for competence in galactose metabolism by growth on minimal plates with 2-deoxy-galactose (DOG) and glycerol as carbon sources. The genome of the double k.o. mutant virus (deficient for *BCRF1* and *BNLF2a*) was generated by conversion of the BNLF2a-Met1 to a STOP codon in the *ΔBCRF1* EBV genome. BAC integrity and the presence of the knockout-specific restriction sites were confirmed by sequencing regions of approx. 5,000 bp around the mutation sites. Primer sequences used for cloning are given in Table S1.

### Generation of virus producer clones and quantification of virus stocks

Recombinant EBV BAC-DNAs were prepared from bacteria, purified on a CsCl gradient and transfected into 293HEK cells using polyethylenimine (Sigma-Aldrich, Munich, Germany). Cells were selected for hygromycin resistance (80 µg/ml, Life Technologies, Darmstadt, Germany) and single clones were tested for GFP fluorescence. Virus production was induced by co-transfection of the expression plasmids p509, encoding *BZLF1* [27], and p2670, encoding *BALF4* [49]. Supernatants were harvested three days later and cells and debris were removed by centrifugation and filtration through PVDF membranes (0.8 µm pore size). Titers of virus stocks (referred to as ‘green Raji units’, GRU) were calculated as described [50] by infecting Raji B cells and measuring the number of GFP+ cells by flow cytometry three days later. Calculated titers were confirmed by infecting Raji cells with equal amounts of GRU from the different virus stocks resulting in equal percentages of GFP+ cells three days p.i. in all samples (Figure S8). Genomic DNA from producer clones was extracted, digested with restriction enzymes and analyzed by Southern blot as described [25]. Blots were hybridized against PCR-amplified DNA fragments derived from EBV's origin of replication, *oriP*, from the *BNLF2b* locus representing sequences adjacent to the *BCRF1* and *BNLF2a* genes, respectively. Primer sequences are provided in Supplementary Table S2.

### Isolation and infection of B cells

Primary B cells were either isolated from PBMCs of voluntary blood donors or buffy coats with the B cell isolation kit II (Miltenyi) yielding B cell populations of ≥95% purity. B cells were infected with EBV mutants at a multiplicity of infection (MOI) of 0.1 GRU/B cell and infected B cells were analyzed for their EBV genotype by PCR amplification of BCRF1, the BNLF2a wild-type sequence or the BNLF2a k.o. (Met1STOP) sequence. The infection with recombinant EBV was confirmed with a PCR spanning GFP and a part of the 2089 EBV backbone. Primer sequences are provided online in Supplementary Table S2.

### T cell recognition assays

The recognition of EBV-infected B cells by EBV-specific CD8+ T cell clones was analyzed by IFN-γ ELISA assays. CD8+ specific T cell clones were established from PBMCs as previously described [51]. EBV-specificity of clonal CD8+ cells was verified by flow cytometry after staining with the respective TCR specific multimer (Proimmune, Oxford, UK) and a CD8-specific antibody (BectonDickinson, Heidelberg, Germany). For recognition assays, triplicates of 10,000 specific CD8+ T cells and 20,000 HLA-matched infected B cells were co-incubated for 18 hours on 96 well cluster plates in a total volume of 200 µl. IFN-γ levels in the supernatants were measured by ELISA (Mabtech, Nacka Strand, Sweden). Sample values were normalized to the IFN-γ level obtained from T cells co-incubated with an established EBV+ lymphoblastoid cell line (LCL). The normalization corrected for putative changes in the performance of the T cells during the time course and inaccuracies in T cell counting.

### Multiplex ELISA

Cytokines in the supernatants of EBV-infected PBMCs were measured with the Th1/Th2 11-plex FlowCytomix Kit (BenderMed Systems, Wien, Austria) according to the manufacturer's instructions.

### Flow cytometry

Cells were washed in PBS, counted and stained with fluorophore-coupled antibodies against human CD3, CD210 (IL10R), CD56, CD4 and MHC class I (anti-human HLA A, B, C clone W6/32) (Biolegend, Netherlands). For intracellular flow cytometry, cells were fixed and permeabilized using the Cytofix/Cytoperm Kit (BD, Germany) and blocked with FCS and TruStain FcX (Biolegend) prior to staining with BNLF2a-specific antibody MVH-8E2 (a kind gift from A. Rickinson, Birmingham) or isotype control. Cells were counterstained with APC-coupled anti-rat IgG F(ab′)2 fragments (Jackson, Newmarket, UK).

### Killing assays

B cells isolated from PBMCs were infected with EBV mutant viruses, cultured for three days and then labeled with calcein (Calcein AM, Life Technologies) as described previously [52]. Autologous CD56+ cells and CD4+ cells were isolated by MACS sorting (Miltenyi, Bergisch-Gladbach, Germany), analyzed by flow cytometry and used for further experiments in case of ≥95% purity. Infected B cells were then co-incubated with CD56+ and/or CD4+ cells in 96-well V-bottomed microtest plates at the indicated effector : target ratios with 1 unit representing 1,000 cells in a total volume of 200 µl. Three hours later, fluorescence in the supernatant was measured with a Wallac Victor plate reader (Perkin-Elmer, Waltham MA, USA). Spontaneous calcein release of labeled cells without effectors was subtracted from sample values. Specific lysis represents the ratio of sample values to total lysis values. Total lysis was obtained by adding 1% Triton-X-100 to target cells.

### Limiting dilutions and regression assays

The transformation capacities of the recombinant viruses were assessed by limiting dilution assays as described [53]. In brief, serial dilutions of EBV mutant viruses were added to 48 replicates of 1×10^5^ B cells prepared from adenoids in 96-well flat bottom plates and incubated for six weeks with weekly supply of fresh culture medium. Living cells were assessed by MTT assays [54]. Regression assays were performed as described elsewhere [48]. Complete PBMCs or PBMCs depleted of CD56+ or CD4+ cells by MACS sorting were infected overnight with EBV at a MOI of 0.1 GRU/B cell. Cells were supplied with fresh culture medium and seeded in 24 replicates on 96-well flat bottom microtest plates in serial dilutions, ranging from 100,000 to 100 cells/well. The number of wells with proliferating cells was assessed by MTT assays after six weeks of culture.

### Expression analysis

B cells from adenoids were infected with EBV mutant viruses at a MOI of 0.1 GRU. Cells were harvested for total RNA preparation at different time points using the RNeasy MiniKit (Qiagen, Hilden, Germany). Residual genomic DNA was removed by DNAse digestion and RNA was reversely transcribed with the SuperMix Kit (Life Technologies). Quantitative PCR was performed on a LightCycler 480 (Roche, Basel, Switzerland), using the SybrGreen LC480 Mix. Primer sequences are provided online in supplementary Table S3.

## Supporting Information

Figure S1 **Genetic manipulation of the 2089 EBV genome.** (A) The 2089 EBV BAC was genetically modified by homologous recombination (see Materials and Methods for details). The BCRF1 gene was replaced by a prokaryotic expression cassette for kanamycin resistance. The translation of BNLF2a was prevented by traceless mutagenesis of the Methionine1-codon to a stop-codon and introduction of a *Spe*I site for analytic purpose. (B) 1 µg of BAC DNAs were digested with the indicated restriction enzymes and separated on an agarose gel. Mutation-specific alterations are indicated for BCRF1 (arrows) and BNLF2a (arrowhead).
(PDF)

Figure S2 **Expression levels of BZLF1, EBNA3a and LMP2AB are not affected by BNLF2a or BCRF1.** Primary B cells were infected with the mutant viruses, total RNA was isolated at different time points post infection and quantitative RT-PCR was performed. Expression levels are shown in relation to transcript levels of the housekeeping gene glucuronidase beta (GUSB). Multiplicities of expression were calculated by normalizing to values from the 2089 EBV-infected samples. Expression of the immediate early gene BZLF1 and the latent genes EBNA3a and LMP2AB did not differ significantly between mutant virus-infected samples and 2089 EBV-infected samples.
(PDF)

Figure S3 **Infection of B cells with mutant EBVs does not affect MHC class I surface levels.** (A) B cells were infected with 2089 EBV or mutant viruses. 2×10^5^ cells of each sample were stained for MHC class I surface expression at the indicated day post infection and analyzed by flow cytometry. MHC I-PerCP mean fluorescence intensities (MFI) were determined for GFP+, i.e. infected, cells. A sample of not infected B cells (not inf.) was analyzed immediately after B cell preparation. (B) 1×10^6^ B cells were analyzed for MHC I surface levels and BNLF2a expression prior to infection and 3 days post infection with the indicated viruses. n.a., not assessed. (C) Same data as in B, shown as density plots.
(PDF)

Figure S4 **Infection of B cells with mutant EBVs did not affect NKG2D-ligand expression levels.** B cells were infected with 2089 EBV or mutant viruses. Total RNA was isolated at the different time points and the transcript levels of the indicated genes were assessed by quantitative RT-PCR. Expression levels are shown in relation to transcript levels of the housekeeping gene glucuronidase beta (*GUSB*). Multiplicities of expression were calculated by normalizing to values from the 2089 EBV-infected samples.
(PDF)

Figure S5 **NK cells, NKT cells and CD4+ T cells express the IL-10 receptor.** 1×10^6^ PBMCs were stained with CD3-FITC, CD56-PE.Cy5, and IL10R-PE or an irrelevant isotype-PE antibody. Cells were gated for lymphocytes and analyzed for IL-10R expression as indicated.
(PDF)

Figure S6 **Exogenous IL-10 rescues the phenotype of BCRF1-deficient EBV mutant viruses.** Killing assays were performed and evaluated as described in Figure 4B. IL-10 was added prior to the addition of target cells to a final concentration of 1 ng/ml. Effector/target ratios were 10∶1 (left panel) and effector/helper/target ratios were 2∶1∶1 (right panel).
(PDF)

Figure S7 **Effects of IL-10 on NK-mediated killing of K562 cells.** Killing assays using K562 cells as targets were performed and evaluated as described in Figure 4B, IL-10 was added to the indicated final concentrations.
(PDF)

Figure S8 **Titration of virus supernatants on Raji cells.** Raji cells were infected with equal volumes of different virus supernatants. The amount of GFP+ Raji cells was assessed by flow cytometry (left panel) and served to calculate the content of ‘green Raji units’ (GRU) per ml of the virus stock. In a second experiment, Raji cells were infected with equal GRUs of the virus stocks and GFP+ cells were assessed on day 3 p.i. (right panel). In case of more than 10% difference in GFP+ cells between the infected samples, titers were corrected and the experiment was repeated.
(PDF)

Figure S9 **Relative and absolute expression of EBV genes.** B cells were isolated from peripheral blood and infected with 2089 EBV. RNA was isolated at indicated timepoints, reversely transcribed and expression levels of indicated genes were determined by qPCR. Panels show expression levels related to the housekeeping gene GUSB and corrected for PCR efficiencies (upper rows) or the second derivative maximum of the fluorescence graph depicted as crossing point (Cp) (lower rows). Values for established cell lines represent expression in 2089 EBV-infected B cells two months after infection ( = LCL) and long-term cultures of the Akata cell line, respectively. Cells were stimulated with anti-human IgA/M/G at 20 µg/ml for 36 hours, LCLs were treated additionally with butyrate (300 µM) and TPA (20 ng/ml). Shown are expression levels of (A) selected antigens, (B) selected immunoevasins and (C) the housekeeping gene GUSB. (D) Numeric Cp values for GUSB transcripts are shown for the indicated time points and samples. Mean values were calculated from three replicates. not inf., not infected; not ind., not induced; n.d., not detected; dpi, days post infection; SD, standard deviation.
(PDF)

Table S1 Found at: doi:Primers for DBNLF2a cloning. This table lists the primers that were used for the generation of the DBNLF2a mutant EBV.
(PDF)

Table S2 **PCR primers.** This table lists the primers that were used for PCR.
(PDF)

Table S3 **qPCR primers.** This table lists the primers that were used for real-time qPCR.
(PDF)

## References

[1] Rickinson A, Kieff E, Knipe D, Howley P (2007). Epstein-Barr Virus.. Fields Virology.

[2] Long HM, Taylor GS, Rickinson AB (2011). Immune defence against EBV and EBV-associated disease.. Curr Opin Immunol.

[3] Kalla M, Schmeinck A, Bergbauer M, Pich D, Hammerschmidt W (2010). AP-1 homolog BZLF1 of Epstein-Barr virus has two essential functions dependent on the epigenetic state of the viral genome.. Proc Natl Acad Sci U S A.

[4] Wen W, Iwakiri D, Yamamoto K, Maruo S, Kanda T (2007). Epstein-Barr virus BZLF1 gene, a switch from latency to lytic infection, is expressed as an immediate-early gene after primary infection of B lymphocytes.. J Virol.

[5] Ressing ME, Horst D, Griffin BD, Tellam J, Zuo J (2008). Epstein-Barr virus evasion of CD8(+) and CD4(+) T cell immunity via concerted actions of multiple gene products.. Semin Cancer Biol.

[6] Miyazaki I, Cheung RK, Dosch HM (1993). Viral interleukin 10 is critical for the induction of B cell growth transformation by Epstein-Barr virus.. J Exp Med.

[7] Salek-Ardakani S, Arrand JR, Mackett M (2002). Epstein-Barr virus encoded interleukin-10 inhibits HLA-class I, ICAM-1, and B7 expression on human monocytes: implications for immune evasion by EBV.. Virology.

[8] Zeidler R, Eissner G, Meissner P, Uebel S, Tampe R (1997). Downregulation of TAP1 in B lymphocytes by cellular and Epstein-Barr virus-encoded interleukin-10.. Blood.

[9] Rode HJ, Bugert JJ, Handermann M, Schnitzler P, Kehm R (1994). Molecular characterization and determination of the coding capacity of the genome of equine herpesvirus type 2 between the genome coordinates 0.235 and 0.258 (the EcoRI DNA fragment N; 4.2 kbp).. Virus Genes.

[10] Zuo J, Thomas W, van Leeuwen D, Middeldorp JM, Wiertz EJ (2008). The DNase of gammaherpesviruses impairs recognition by virus-specific CD8+ T cells through an additional host shutoff function.. J Virol.

[11] van Gent M, Griffin BD, Berkhoff EG, van Leeuwen D, Boer IG (2011). EBV lytic-phase protein BGLF5 contributes to TLR9 downregulation during productive infection.. J Immunol.

[12] Zuo J, Quinn LL, Tamblyn J, Thomas WA, Feederle R (2011). The Epstein-Barr virus-encoded BILF1 protein modulates immune recognition of endogenously processed antigen by targeting major histocompatibility complex class I molecules trafficking on both the exocytic and endocytic pathways.. J Virol.

[13] Hislop AD, Ressing ME, van Leeuwen D, Pudney VA, Horst D (2007). A CD8+ T cell immune evasion protein specific to Epstein-Barr virus and its close relatives in Old World primates.. J Exp Med.

[14] Haig DM, Fleming S (1999). Immunomodulation by virulence proteins of the parapoxvirus orf virus.. Vet Immunol Immunopathol.

[15] Kotenko SV, Saccani S, Izotova LS, Mirochnitchenko OV, Pestka S (2000). Human cytomegalovirus harbors its own unique IL-10 homolog (cmvIL-10).. Proc Natl Acad Sci U S A.

[16] Raftery MJ, Wieland D, Gronewald S, Kraus AA, Giese T (2004). Shaping phenotype, function, and survival of dendritic cells by cytomegalovirus-encoded IL-10.. J Immunol.

[17] Spencer JV, Lockridge KM, Barry PA, Lin G, Tsang M (2002). Potent immunosuppressive activities of cytomegalovirus-encoded interleukin-10.. J Virol.

[18] Chang WL, Barry PA (2010). Attenuation of innate immunity by cytomegalovirus IL-10 establishes a long-term deficit of adaptive antiviral immunity.. Proc Natl Acad Sci U S A.

[19] Swaminathan S, Hesselton R, Sullivan J, Kieff E (1993). Epstein-Barr virus recombinants with specifically mutated BCRF1 genes.. J Virol.

[20] Bejarano MT, Masucci MG (1998). Interleukin-10 abrogates the inhibition of Epstein-Barr virus-induced B-cell transformation by memory T-cell responses.. Blood.

[21] Parcej D, Tampe R (2010). ABC proteins in antigen translocation and viral inhibition.. Nat Chem Biol.

[22] Horst D, van Leeuwen D, Croft NP, Garstka MA, Hislop AD (2009). Specific targeting of the EBV lytic phase protein BNLF2a to the transporter associated with antigen processing results in impairment of HLA class I-restricted antigen presentation.. J Immunol.

[23] Ljunggren HG, Stam NJ, Ohlen C, Neefjes JJ, Hoglund P (1990). Empty MHC class I molecules come out in the cold.. Nature.

[24] Croft NP, Shannon-Lowe C, Bell AI, Horst D, Kremmer E (2009). Stage-specific inhibition of MHC class I presentation by the Epstein-Barr virus BNLF2a protein during virus lytic cycle.. PLoS Pathog.

[25] Delecluse HJ, Hilsendegen T, Pich D, Zeidler R, Hammerschmidt W (1998). Propagation and recovery of intact, infectious Epstein-Barr virus from prokaryotic to human cells.. Proc Natl Acad Sci U S A.

[26] Warming S, Costantino N, Court DL, Jenkins NA, Copeland NG (2005). Simple and highly efficient BAC recombineering using galK selection.. Nucleic Acids Res.

[27] Hammerschmidt W, Sugden B (1988). Identification and characterization of oriLyt, a lytic origin of DNA replication of Epstein-Barr virus.. Cell.

[28] Jochum S, Ruiss R, Hammerschmidt W, Zeidler R (2012). Functional viral RNAs in Epstein-Barr-Virus particles.. Proc Natl Acad Sci U S A.

[29] de Brouwer AP, van Bokhoven H, Kremer H (2006). Comparison of 12 reference genes for normalization of gene expression levels in Epstein-Barr virus-transformed lymphoblastoid cell lines and fibroblasts.. Mol Diagn Ther.

[30] Elliott SL, Pye SJ, Schmidt C, Cross SM, Silins SL (1997). Dominant cytotoxic T lymphocyte response to the immediate-early trans-activator protein, BZLF1, in persistent type A or B Epstein-Barr virus infection.. J Infect Dis.

[31] Countryman J, Miller G (1985). Activation of expression of latent Epstein-Barr herpesvirus after gene transfer with a small cloned subfragment of heterogeneous viral DNA.. Proc Natl Acad Sci U S A.

[32] Lautscham G, Mayrhofer S, Taylor G, Haigh T, Leese A (2001). Processing of a multiple membrane spanning Epstein-Barr virus protein for CD8(+) T cell recognition reveals a proteasome-dependent, transporter associated with antigen processing-independent pathway.. J Exp Med.

[33] Subklewe M, Sebelin K, Block A, Meier A, Roukens A (2005). Dendritic cells expand Epstein Barr virus specific CD8+ T cell responses more efficiently than EBV transformed B cells.. Hum Immunol.

[34] Pudney VA, Leese AM, Rickinson AB, Hislop AD (2005). CD8+ immunodominance among Epstein-Barr virus lytic cycle antigens directly reflects the efficiency of antigen presentation in lytically infected cells.. J Exp Med.

[35] Kanai K, Satoh Y, Yamanaka H, Kawaguchi A, Horie K (2007). The vIL-10 gene of the Epstein-Barr virus (EBV) is conserved in a stable manner except for a few point mutations in various EBV isolates.. Virus Genes.

[36] Moore KW, de Waal Malefyt R, Coffman RL, O'Garra A (2001). Interleukin-10 and the interleukin-10 receptor.. Annu Rev Immunol.

[37] Pappworth IY, Wang EC, Rowe M (2007). The switch from latent to productive infection in epstein-barr virus-infected B cells is associated with sensitization to NK cell killing.. J Virol.

[38] Serrano AE, Menares-Castillo E, Garrido-Tapia M, Ribeiro CH, Hernandez CJ (2011). Interleukin 10 decreases MICA expression on melanoma cell surface.. Immunol Cell Biol.

[39] Carson WE, Lindemann MJ, Baiocchi R, Linett M, Tan JC (1995). The functional characterization of interleukin-10 receptor expression on human natural killer cells.. Blood.

[40] Frisan T, Levitsky V, Masucci M (2001). Limiting dilution assay.. Methods Mol Biol.

[41] Gudgeon NH, Taylor GS, Long HM, Haigh TA, Rickinson AB (2005). Regression of Epstein-Barr virus-induced B-cell transformation in vitro involves virus-specific CD8+ T cells as the principal effectors and a novel CD4+ T-cell reactivity.. J Virol.

[42] Janssen EM, Lemmens EE, Wolfe T, Christen U, von Herrath MG (2003). CD4+ T cells are required for secondary expansion and memory in CD8+ T lymphocytes.. Nature.

[43] Shedlock DJ, Shen H (2003). Requirement for CD4 T cell help in generating functional CD8 T cell memory.. Science.

[44] Steven NM, Annels NE, Kumar A, Leese AM, Kurilla MG (1997). Immediate early and early lytic cycle proteins are frequent targets of the Epstein-Barr virus-induced cytotoxic T cell response.. J Exp Med.

[45] Kastenmuller W, Gasteiger G, Gronau JH, Baier R, Ljapoci R (2007). Cross-competition of CD8+ T cells shapes the immunodominance hierarchy during boost vaccination.. J Exp Med.

[46] Kurth J, Spieker T, Wustrow J, Strickler GJ, Hansmann LM (2000). EBV-infected B cells in infectious mononucleosis: viral strategies for spreading in the B cell compartment and establishing latency.. Immunity.

[47] Taga H, Taga K, Wang F, Chretien J, Tosato G (1995). Human and viral interleukin-10 in acute Epstein-Barr virus-induced infectious mononucleosis.. J Infect Dis.

[48] Rickinson AB, Rowe M, Hart IJ, Yao QY, Henderson LE (1984). T-cell-mediated regression of “spontaneous” and of Epstein-Barr virus-induced B-cell transformation in vitro: studies with cyclosporin A.. Cell Immunol.

[49] Neuhierl B, Feederle R, Hammerschmidt W, Delecluse HJ (2002). Glycoprotein gp110 of Epstein-Barr virus determines viral tropism and efficiency of infection.. Proc Natl Acad Sci U S A.

[50] Altmann M, Hammerschmidt W (2005). Epstein-Barr virus provides a new paradigm: a requirement for the immediate inhibition of apoptosis.. PLoS Biol.

[51] Moosmann A, Khan N, Cobbold M, Zentz C, Delecluse HJ (2002). B cells immortalized by a mini-Epstein-Barr virus encoding a foreign antigen efficiently reactivate specific cytotoxic T cells.. Blood.

[52] Wiesner M, Zentz C, Mayr C, Wimmer R, Hammerschmidt W (2008). Conditional immortalization of human B cells by CD40 ligation.. PLoS One.

[53] Wilson JB May GHW 2001 Epstein-Barr virus protocols Totowa, N.J. Humana Press xiv, 438

[54] Cory AH, Owen TC, Barltrop JA, Cory JG (1991). Use of an aqueous soluble tetrazolium/formazan assay for cell growth assays in culture.. Cancer Commun.

