# Supplementary figures and images for: The EBV Immunoevasins vIL-10 and BNLF2a Protect Newly Infected B Cells from Immune Recognition and Elimination
Source: PLoS Pathog. 2012 May 17;8(5):e1002704. doi: 10.1371/journal.ppat.1002704 (PMC3355093; doi:10.1371/journal.ppat.1002704)

A.

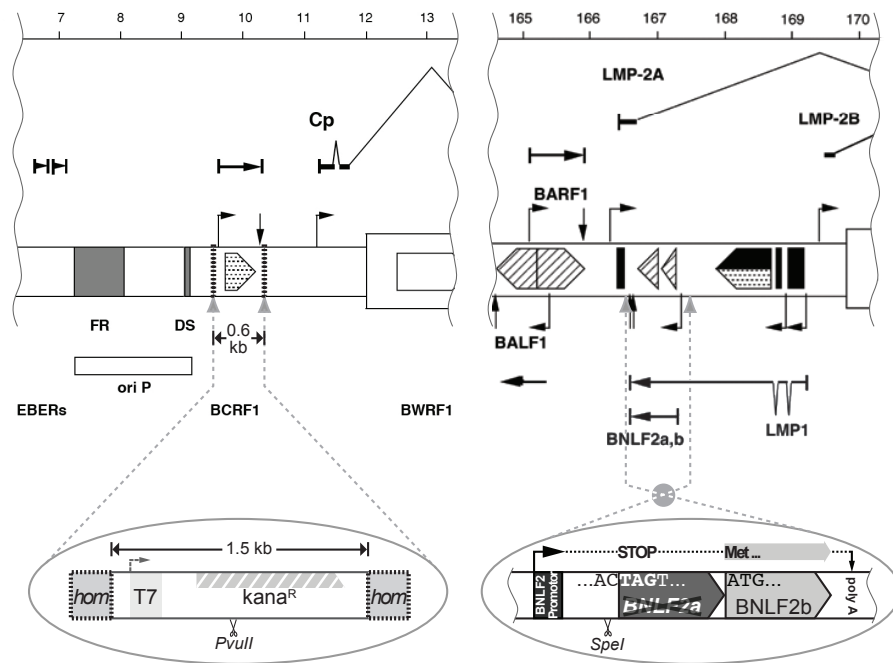

B.

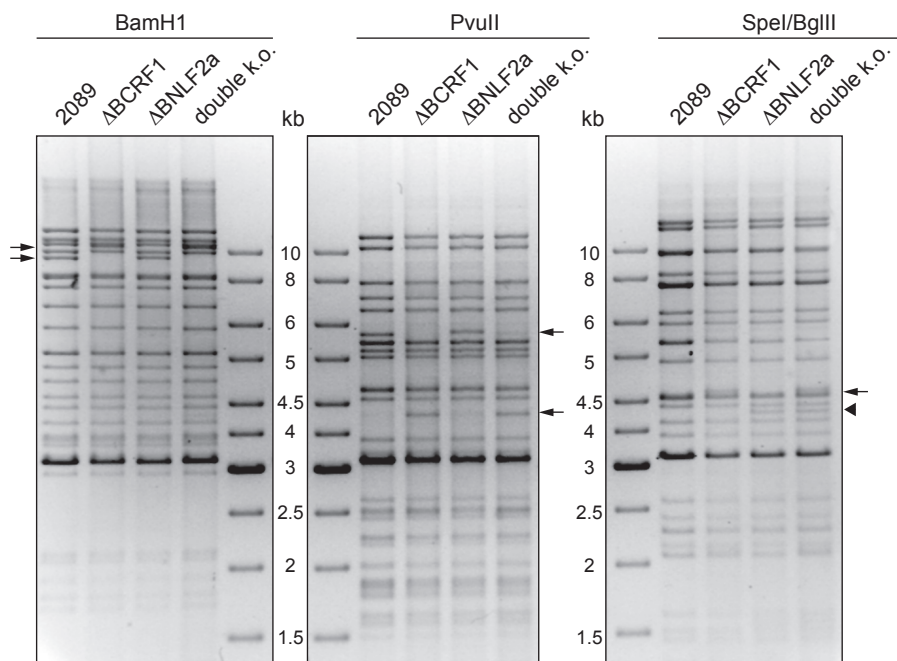

## Figure S1

Supplement: Figure S1 — Genetic manipulation of the 2089 EBV genome. (A) The 2089 EBV BAC was genetically modified by homologous recombination (see Materials and Methods for details). The BCRF1 gene was replaced by a prokaryotic expression cassette for kanamycin resistance. The translation of BNLF2a was prevented by traceless mutagenesis of the Methionine1-codon to a stop-codon and introduction of a SpeI site for analytic purpose. (B) 1 µg of BAC DNAs were digested with the indicated restriction enzymes and separated on an agarose gel. Mutation-specific alterations are indicated for BCRF1 (arrows) and BNLF2a (arrowhead). (PDF) [file ppat.1002704.s001.pdf]

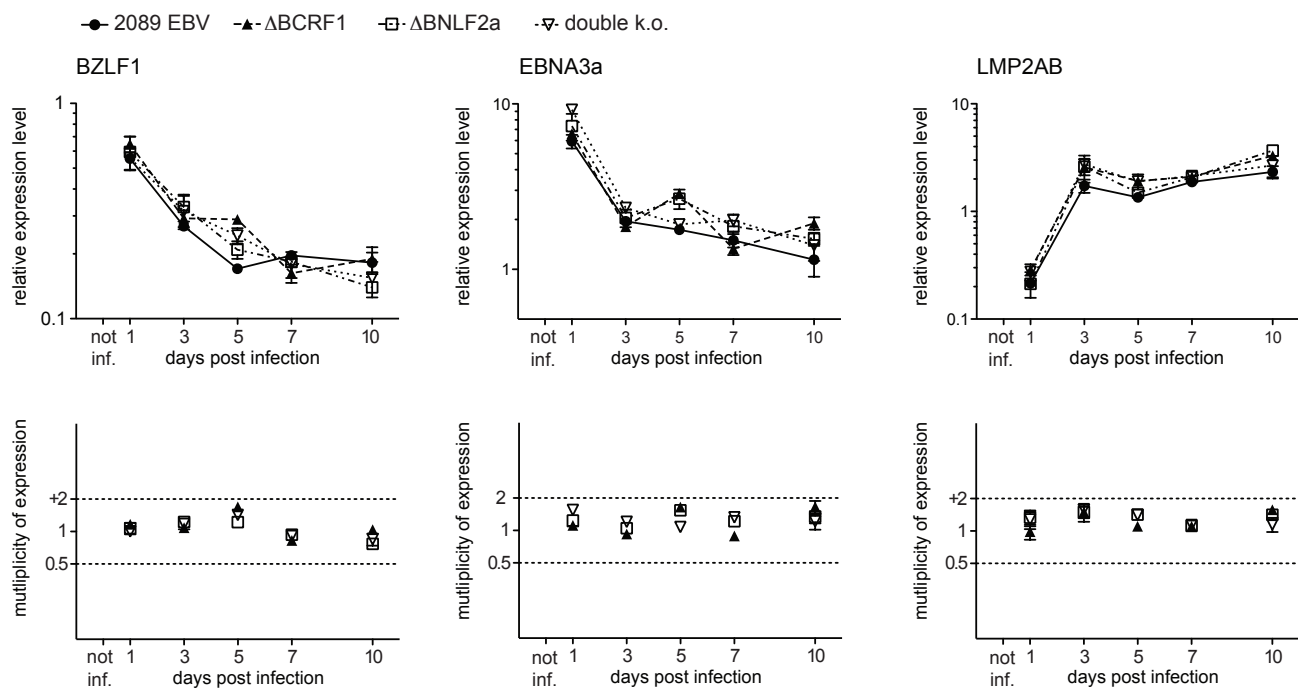

Figure S2

Supplement: Figure S2 — Expression levels of BZLF1, EBNA3a and LMP2AB are not affected by BNLF2a or BCRF1. Primary B cells were infected with the mutant viruses, total RNA was isolated at different time points post infection and quantitative RT-PCR was performed. Expression levels are shown in relation to transcript levels of the housekeeping gene glucuronidase beta (GUSB). Multiplicities of expression were calculated by normalizing to values from the 2089 EBV-infected samples. Expression of the immediate early gene BZLF1 and the latent genes EBNA3a and LMP2AB did not differ significantly between mutant virus-infected samples and 2089 EBV-infected samples. (PDF) [file ppat.1002704.s002.pdf]

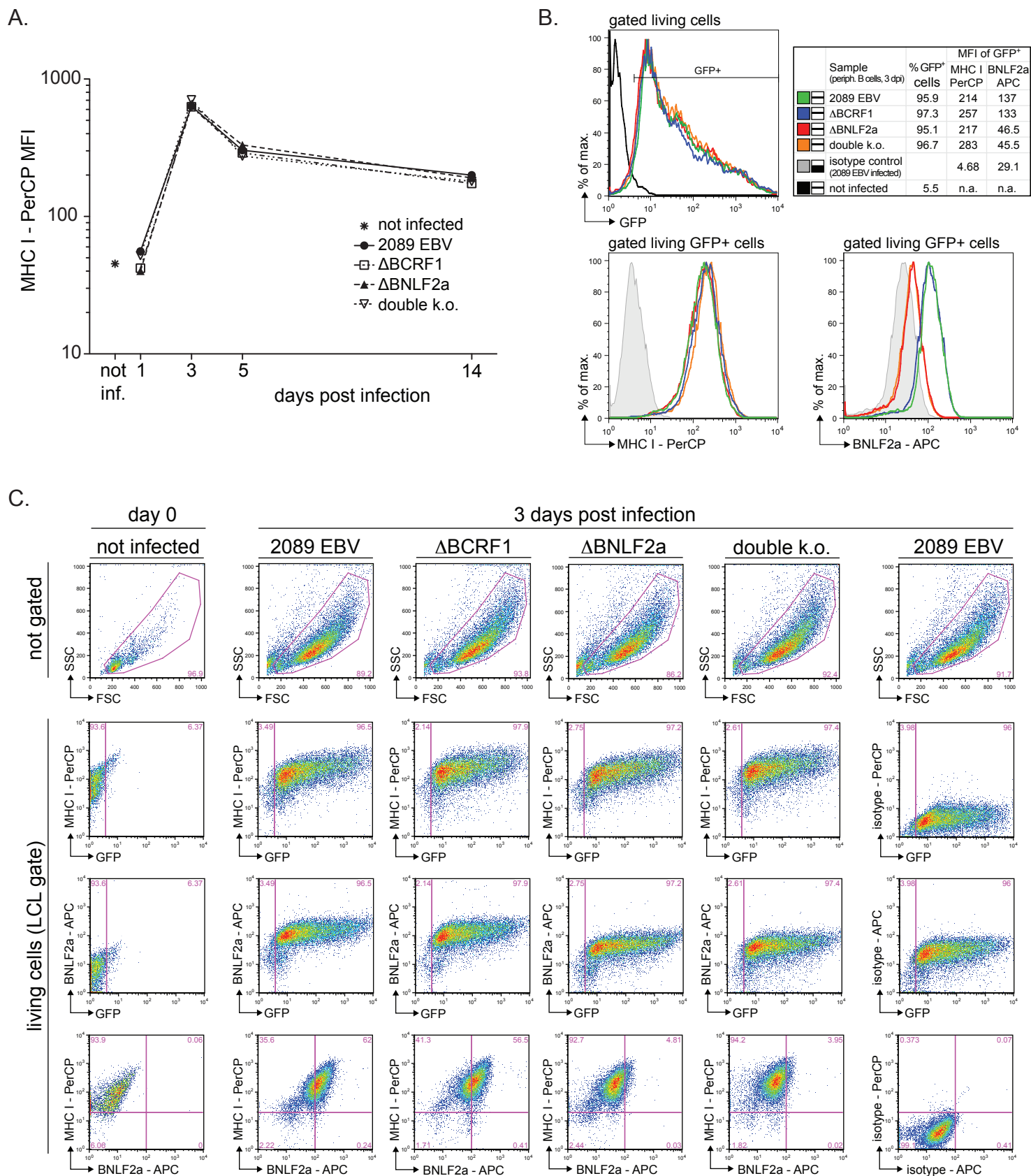

Figure S3

Supplement: Figure S3 — Infection of B cells with mutant EBVs does not affect MHC class I surface levels. (A) B cells were infected with 2089 EBV or mutant viruses. 2×10^5^ cells of each sample were stained for MHC class I surface expression at the indicated day post infection and analyzed by flow cytometry. MHC I-PerCP mean fluorescence intensities (MFI) were determined for GFP+, i.e. infected, cells. A sample of not infected B cells (not inf.) was analyzed immediately after B cell preparation. (B) 1×10^6^ B cells were analyzed for MHC I surface levels and BNLF2a expression prior to infection and 3 days post infection with the indicated viruses. n.a., not assessed. (C) Same data as in B, shown as density plots. (PDF) [file ppat.1002704.s003.pdf]

\* not infected    ● 2089 EBV    □ ΔBCRF1    ▲ ΔBNLF2a    ▽ double k.o.

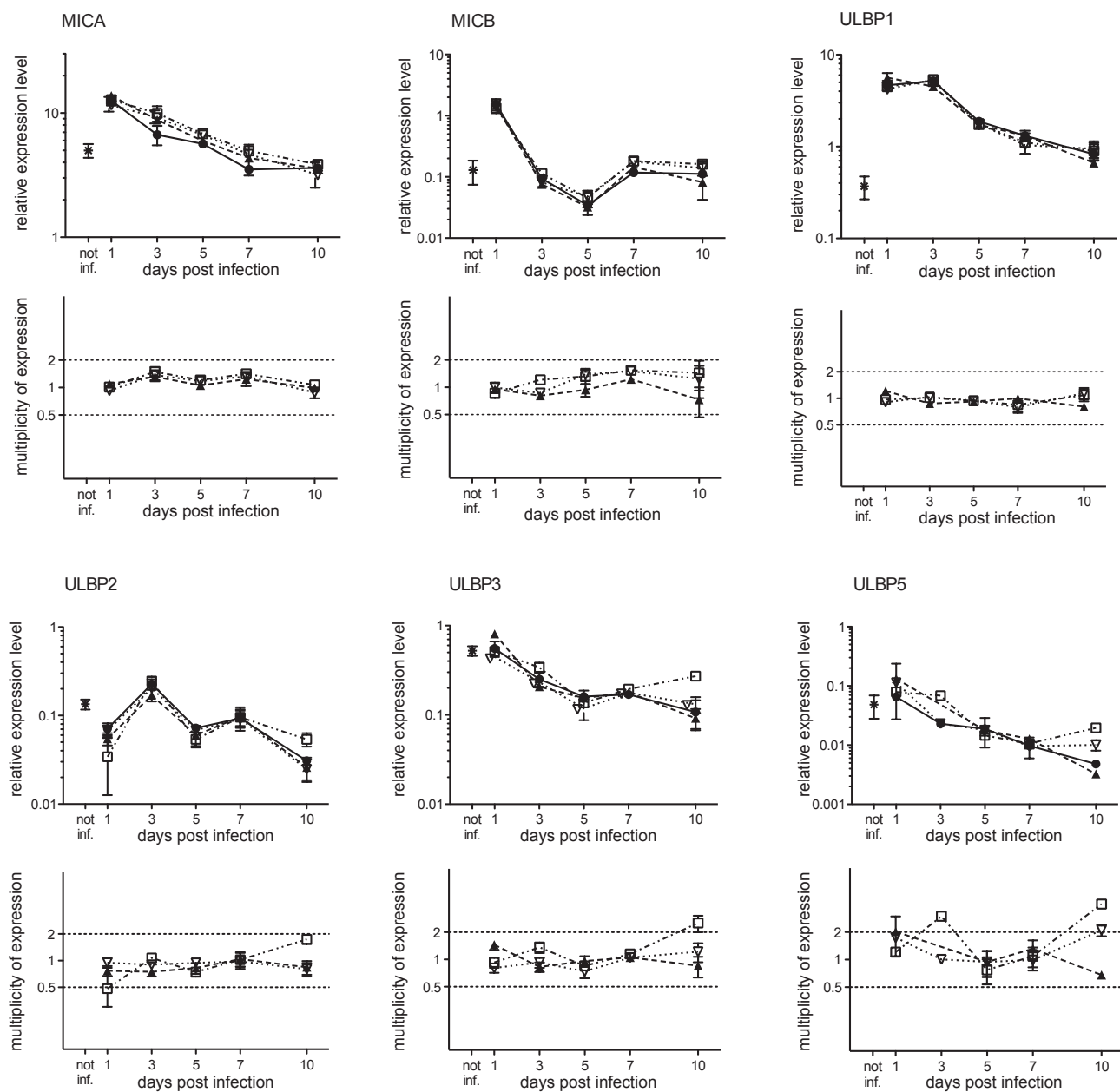

Figure S4

Supplement: Figure S4 — Infection of B cells with mutant EBVs did not affect NKG2D-ligand expression levels. B cells were infected with 2089 EBV or mutant viruses. Total RNA was isolated at the different time points and the transcript levels of the indicated genes were assessed by quantitative RT-PCR. Expression levels are shown in relation to transcript levels of the housekeeping gene glucuronidase beta (GUSB). Multiplicities of expression were calculated by normalizing to values from the 2089 EBV-infected samples. (PDF) [file ppat.1002704.s004.pdf]

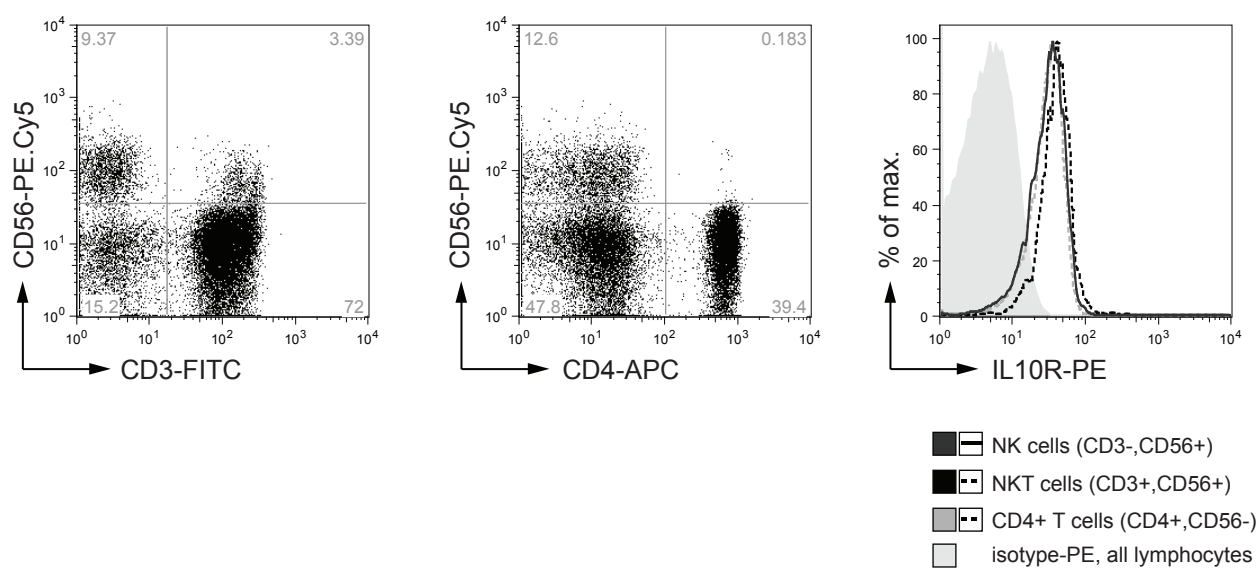

Figure S5

Supplement: Figure S5 — NK cells, NKT cells and CD4+ T cells express the IL-10 receptor. 1×10^6^ PBMCs were stained with CD3-FITC, CD56-PE.Cy5, and IL10R-PE or an irrelevant isotype-PE antibody. Cells were gated for lymphocytes and analyzed for IL-10R expression as indicated. (PDF) [file ppat.1002704.s005.pdf]

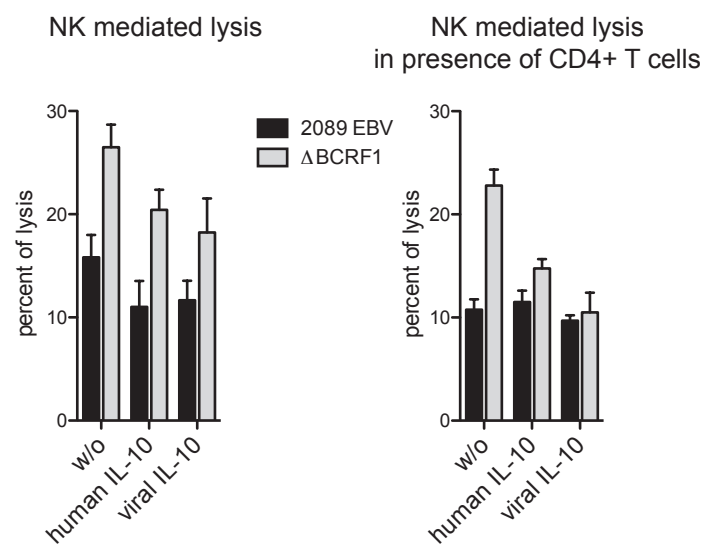

Figure S6

Supplement: Figure S6 — Exogenous IL-10 rescues the phenotype of BCRF1-deficient EBV mutant viruses. Killing assays were performed and evaluated as described in Figure 4B. IL-10 was added prior to the addition of target cells to a final concentration of 1 ng/ml. Effector/target ratios were 10∶1 (left panel) and effector/helper/target ratios were 2∶1∶1 (right panel). (PDF) [file ppat.1002704.s006.pdf]

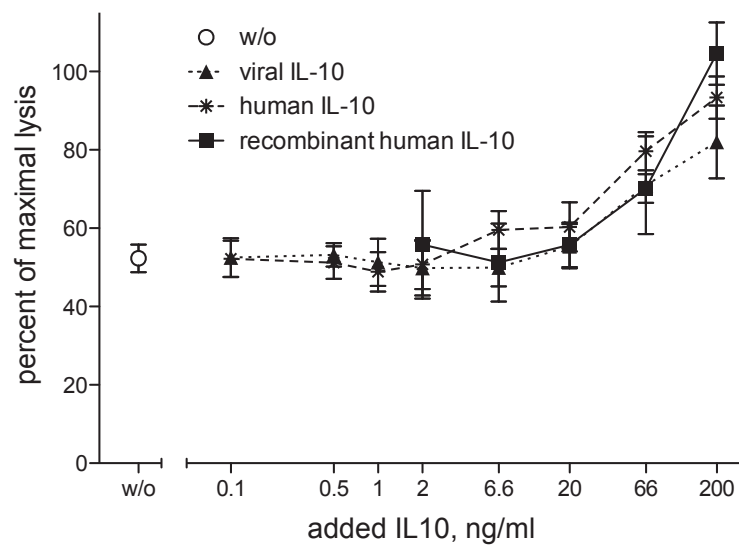

Figure S7

Supplement: Figure S7 — Effects of IL-10 on NK-mediated killing of K562 cells. Killing assays using K562 cells as targets were performed and evaluated as described in Figure 4B, IL-10 was added to the indicated final concentrations. (PDF) [file ppat.1002704.s007.pdf]

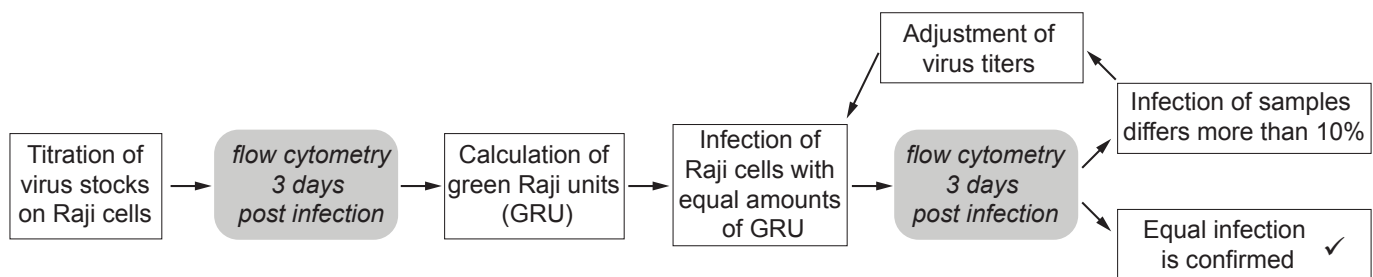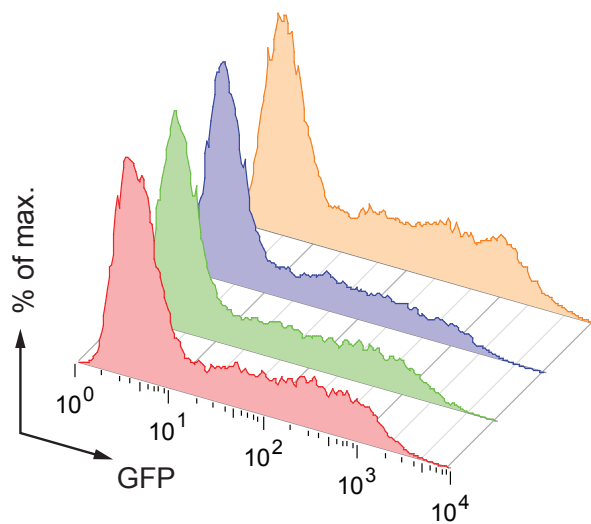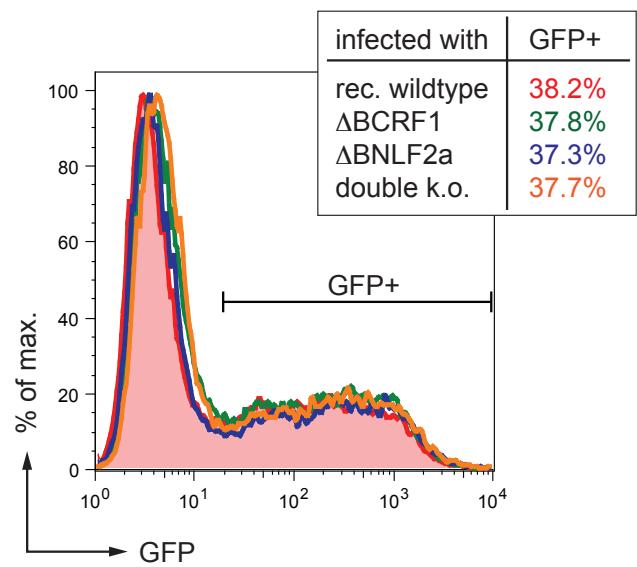

Figure S8

Supplement: Figure S8 — Titration of virus supernatants on Raji cells. Raji cells were infected with equal volumes of different virus supernatants. The amount of GFP+ Raji cells was assessed by flow cytometry (left panel) and served to calculate the content of ‘green Raji units’ (GRU) per ml of the virus stock. In a second experiment, Raji cells were infected with equal GRUs of the virus stocks and GFP+ cells were assessed on day 3 p.i. (right panel). In case of more than 10% difference in GFP+ cells between the infected samples, titers were corrected and the experiment was repeated. (PDF) [file ppat.1002704.s008.pdf]
