# Supplementary material for: The EBV Immunoevasins vIL-10 and BNLF2a Protect Newly Infected B Cells from Immune Recognition and Elimination
Source: PLoS Pathog. 2012 May 17;8(5):e1002704. doi: 10.1371/journal.ppat.1002704 (PMC3355093; doi:10.1371/journal.ppat.1002704)
Supplement: Figure S9 — Relative and absolute expression of EBV genes. B cells were isolated from peripheral blood and infected with 2089 EBV. RNA was isolated at indicated timepoints, reversely transcribed and expression levels of indicated genes were determined by qPCR. Panels show expression levels related to the housekeeping gene GUSB and corrected for PCR efficiencies (upper rows) or the second derivative maximum of the fluorescence graph depicted as crossing point (Cp) (lower rows). Values for established cell lines represent expression in 2089 EBV-infected B cells two months after infection ( = LCL) and long-term cultures of the Akata cell line, respectively. Cells were stimulated with anti-human IgA/M/G at 20 µg/ml for 36 hours, LCLs were treated additionally with butyrate (300 µM) and TPA (20 ng/ml). Shown are expression levels of (A) selected antigens, (B) selected immunoevasins and (C) the housekeeping gene GUSB. (D) Numeric Cp values for GUSB transcripts are shown for the indicated time points and samples. Mean values were calculated from three replicates. not inf., not infected; not ind., not induced; n.d., not detected; dpi, days post infection; SD, standard deviation. (PDF) [file ppat.1002704.s009.pdf]

2089 EBV-infected peripheral B cells / LCL — Akata

A.

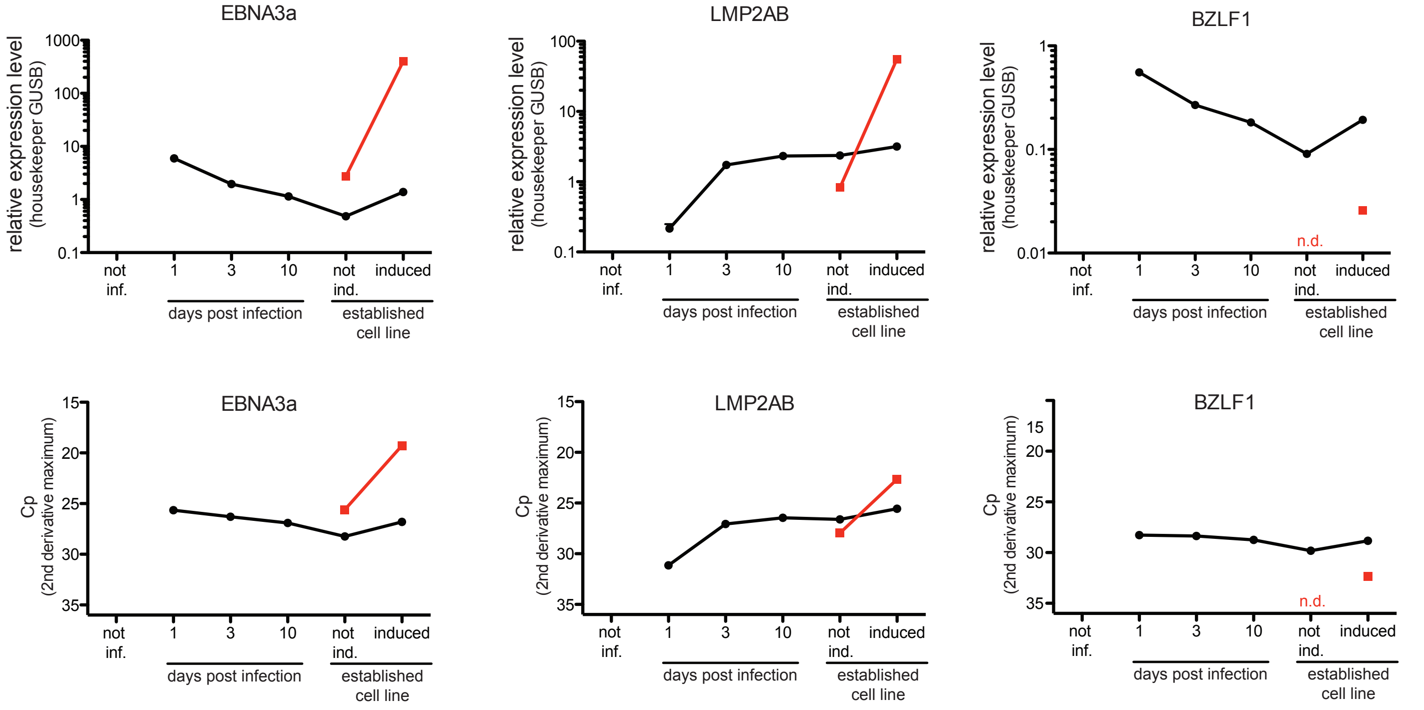

B.

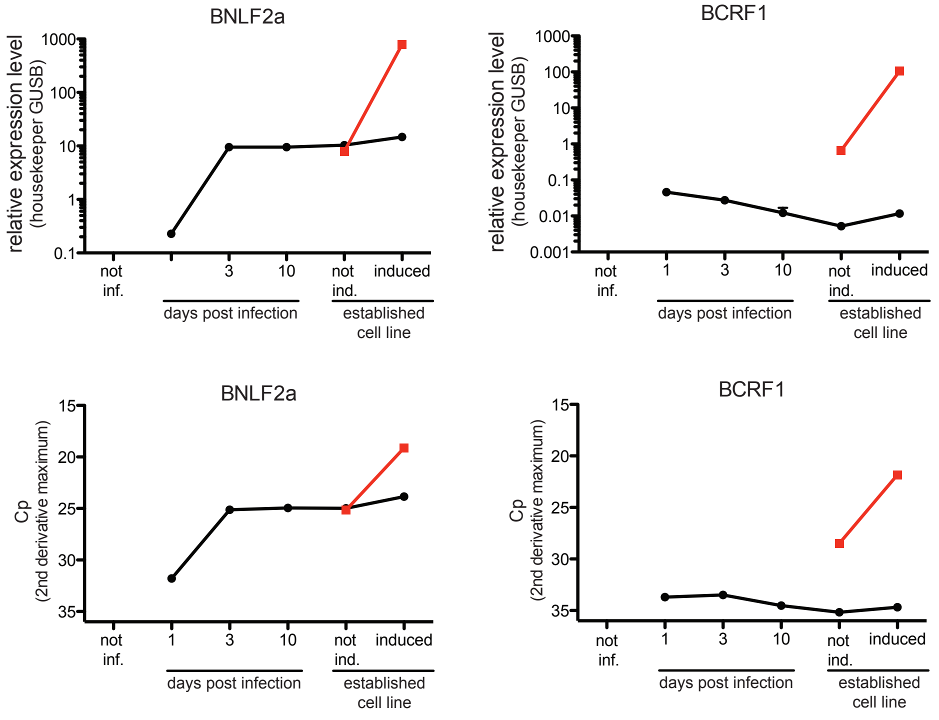

C.

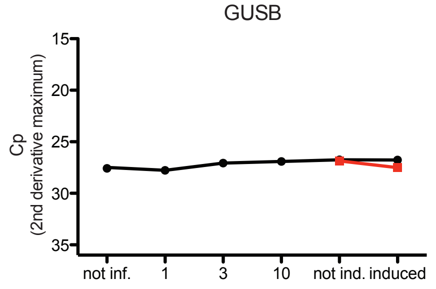

D.

dpi	not infected		2089 EBV	
	Mean	*SD*	*Mean*	*SD*
0	27.52	0.03		
1			27.74	0.04
3			26.69	0.08
5			26.64	0.08
7			27.01	0.05
10			26.91	0.07
LCL			26.79	0.04
induced LCL			26.51	0.07

Figure S9
