# Supplementary material for: The EBV Immunoevasins vIL-10 and BNLF2a Protect Newly Infected B Cells from Immune Recognition and Elimination
Source: PLoS Pathog. 2012 May 17;8(5):e1002704. doi: 10.1371/journal.ppat.1002704 (PMC3355093; doi:10.1371/journal.ppat.1002704)
Supplement: Table S1 — Found at: doi:Primers for DBNLF2a cloning. This table lists the primers that were used for the generation of the DBNLF2a mutant EBV. (PDF) [file ppat.1002704.s010.pdf]

Replacement		Sequence (5’-3’)
BNLF2aSTART → BNLF2aGalK	fw.	^{177267}**CCGCAGGCAGAGGACTGCTGCTCTAGCAAAGCACGCTCCAGGACGTGTA**^{177316} *CCTGTTGACAATTAATCATCGGCA*
	rev.	^{177372}**TGTCCTTACTGTATAAAAGTCCACGAAAACAGCTGTGCCTCACTCTCGA**^{177322} *TCAGCACTGTCCTGCTCCTT*
BNLF2aGalk → BNLF2aSTOP	fw.	^{177258}**GAAGAGCCGGGCAGGCCGCAGGCAGAGGACTGCTGCTCTAGCAAAGCACGCT CCAGGACGTGTA**^{177316}ACTAG^{177322}**TCGAGAGTG**
	rev.	^{177385}**CTGGCCTTCTTTCCTTGTCCTTACTGTATAAAAGTCCACGAAAACAGCTGTG CCTCACTCTCGA**^{177322}CTAGT^{177316}**TACACGTCCT**

bold: homology to the EBV genome; superscripted numbers: genomic position of homologous sequence in p2089; *italic*: matching sequence in PCR template; underlined: complementary region of primer pair.

Table S1
