# Supplementary material for: The EBV Immunoevasins vIL-10 and BNLF2a Protect Newly Infected B Cells from Immune Recognition and Elimination
Source: PLoS Pathog. 2012 May 17;8(5):e1002704. doi: 10.1371/journal.ppat.1002704 (PMC3355093; doi:10.1371/journal.ppat.1002704)
Supplement: Table S2 — PCR primers. This table lists the primers that were used for PCR. (PDF) [file ppat.1002704.s011.pdf]

PCR primers

Target (genomic)	forward primer (5'-3')	reverse primer (5'-3')	product size(bp)
oriP	GTCTTGGTCCCTGCCTGG	GGTTAGTAAAAGGGTCCTAAGGA[illegible]	528
BNLF2b	ACGATGGCGGAAACAACTC	TCCAAAAGGTCAAAGAACAAGG	604
BNLF2a wt	GAGCAGGCATAAAAGTCCAA	GCCTCACTCTCGAGATGG	188
BNLF2a k.o.	GAGCAGGCATAAAAGTCCAA	CTGTGCCTCACTCTCGACTAGT	192
BCRF1	TGGAGCGAAGGTTAGTGGTCA[illegible]	ATGGTCTTTGGCTTCAGGGTCC	300
2089 genome	ATCGACGTATCGCTGGAAAC	CTTGTATGGAGCAGCAGACG	742

Table S2
