# Supplementary material for: The EBV Immunoevasins vIL-10 and BNLF2a Protect Newly Infected B Cells from Immune Recognition and Elimination
Source: PLoS Pathog. 2012 May 17;8(5):e1002704. doi: 10.1371/journal.ppat.1002704 (PMC3355093; doi:10.1371/journal.ppat.1002704)
Supplement: Table S3 — qPCR primers. This table lists the primers that were used for real-time qPCR. (PDF) [file ppat.1002704.s012.pdf]

qPCR primers

Target (transcript)	forward primer (5’-3’)	reverse primer (5’-3’)	product size(bp)
BZLF1	CTGGTGTCCGGGGGATAAT	TCCGCAGGTGGCTGCT	107
BNLF2a	TGCTGACGTCTGGGTCCT	TGCTTTGCTAGAGCAGCAGT	98
BCRF1	ACCTTAGGTATGGAGCGAAG	GGGAAAATTGTCACATTGGT	110
LMP2AB	ATCGCTGGTGGCAGTATTTT	GAGTATGCCAGCGACAATCA	105
EBNA3a	TCCGCAGGTTTCCACTAGAT	GGGATCCGAAAAACTGGTCT	99
GUSB	CGCCCTGCCTATCTGTATTC	TCCCCACAGGGAGTGTGTAG	91
MICA	GCCCTCTGGGAAAGTGCTGGT	AGCAGCAGCAACAGCAGAAACA	70
MICB	TGACCAGCCCAGGACCAGCT	AGAGTAATGCATAACGGTGATCGG	72
ULBP 1	TCCCTGGAGCCTTCTCATC	CTTCTTGATATCACTTTCCATG	95
ULBP 2	CCGCTACCAAGATCCTTCTG	TGACGGTGATGCTATAGCAAA	105
ULBP 3	AGGAAGAAGAGGCTGGAACC	CTATGGCTTTGGGTTGAGACT	70
ULBP 4	TCCTGGGGGCATTCATCCTG	GCCAGAGACCAGCCTGCCAC	90
ULBP 5	ACTTCTTGATGGGCATGGAC	AGGACATGGTGGGTGGTG	63
ULBP 6	CAGCTTTGCTTCTGTGCCCTC	GGTCTGAACTTAGGATGAGTGA	115

Table S3
